# Decoding immune-metabolic crosstalk in ARDS: a transcriptomic exploration of biomarkers, cellular dynamics, and therapeutic pathways

**DOI:** 10.3389/fimmu.2025.1615748

**Published:** 2025-10-27

**Authors:** Ting Wu, Yuan Lu, Qin Wang, Wei Zhou, Ming Ding, Jing Huang, Jingyuan Xu, Shuzhen Wei, Min Wang

**Affiliations:** ^1^ Department of Respiratory and Critical Care Medicine, Zhongda Hospital Southeast University, Nanjing, China; ^2^ Department of Respiratory and Critical Care Medicine, Jinling Hospital, Nanjing University School of Medicine, Nanjing, China; ^3^ Jiangsu Provincial Key Laboratory of Critical Care Medicine, Department of Critical Care Medicine, Zhongda Hospital Southeast University, Nanjing, China; ^4^ General Ward, Shazhou Community Health Service Center, Nanjing, China; ^5^ Department of Respiratory and Critical Care Medicine, The First Affiliated Hospital, and College of Clinical Medicine of Henan University of Science and Technology, Luoyang, China

**Keywords:** acute respiratory distress syndrome, immune cells, metabolic reprogramming, biomarkers, single-cell RNA sequencing

## Abstract

**Background:**

Metabolic reprogramming plays a critical role in various diseases, with particular emphasis on immune cell metabolism. However, the involvement of immune cells and metabolic reprogramming-related genes (MRRGs) in acute respiratory distress syndrome (ARDS) remains underexplored. This study aimed to investigate the molecular mechanisms underlying cell and metabolic reprogramming biomarkers in ARDS.

**Methods:**

Using transcriptomic data from whole blood samples, candidate genes were identified through differential expression analysis and weighted gene co-expression network analysis (WGCNA) in conjunction with MRRGs. Machine learning techniques, expression analysis, and receiver operating characteristic (ROC) analysis were employed to identify potential biomarkers. An artificial neural network (ANN) model was developed and evaluated. Additionally, functional enrichment, regulatory network, and drug prediction analyses were performed. Single-cell analysis was conducted to examine the expression of biomarkers within specific cell populations. Reverse transcription-quantitative polymerase chain reaction (RT-qPCR) was used for biomarker validation in human whole blood samples. The functional validation of candidate biomarkers was performed in lipopolysaccharide (LPS)-induced ARDS mouse models (peripheral blood neutrophils and lung tissues) and THP-1-derived macrophages.

**Results:**

Through machine learning algorithms, RPL14, SMARCD3, and TCN1 were identified as candidate biomarkers. ROC analysis demonstrated that the ANN model, incorporating these biomarkers, exhibited strong predictive power for ARDS onset. Enrichment analysis revealed that these genes were linked to various pathways, including the chemokine signaling pathway. The regulatory network analysis suggested that KLF9 may regulate both RPL14 and SMARCD3, with these genes playing a pivotal role in ARDS progression. Furthermore, selenium (CTD 00006731) and Cyclosporine A(CsA)(CTD 00007121) were identified as compounds targeting RPL14 and SMARCD3. Expression levels of the biomarkers varied across different stages of cell differentiation. RT-qPCR confirmed a significant upregulation of SMARCD3 and TCN1 in ARDS samples, aligning with dataset expression analysis results. Both *in vitro* and *in vivo* experiments demonstrated that modulation of SMARCD3 and TCN1 (but not RPL14) significantly affected mitochondrial function, oxidative stress, apoptosis, glucose metabolism and inflammatory cytokine expression.

**Conclusion:**

SMARCD3 and TCN1 were identified as key biomarkers associated with immune cell and metabolic reprogramming in ARDS, while RPL14 was identified as a candidate biomarker through computational approaches, offering valuable insights for understanding the pathogenesis of the disease.

## 1 Introduction

Acute respiratory distress syndrome (ARDS) is a critical respiratory condition characterized by acute hypoxemic respiratory failure and bilateral infiltrates visible on chest imaging, with no full explanation by cardiac failure or fluid overload (1–3). Clinically, ARDS manifests as severe dyspnea, rapid breathing, and hypoxemia, often progressing to multiple organ failure and a high mortality rate, particularly in critically ill patients (2, 4). This syndrome is associated with significant long-term consequences, including physical, cognitive, and psychological impairments, highlighting its devastating effect on patients’ quality of life (1, 3). Current therapeutic strategies primarily focus on supportive care, such as lung-protective ventilation, prone positioning, and fluid balance management. Despite these measures, treatment outcomes remain suboptimal, with a mortality rate of 30-40% (1–3). Thus, understanding the pathophysiology of ARDS and identifying reliable biomarkers are essential for uncovering potential therapeutic targets.

The pathogenesis of ARDS involves a complex interaction between inflammatory responses, endothelial and epithelial injury, and dysregulated lung inflammation, immune cell activation, and metabolic reprogramming in the lung microenvironment (5–9). These processes lead to increased alveolar-capillary permeability and pulmonary edema (1, 2). Key immune cells, including neutrophils, alveolar macrophages, T-lymphocytes, complement system components, dendritic cells, and NK cells, are central to the disease’s pathophysiology, contributing to both tissue damage and repair (10). Neutrophils, as the initial responders, migrate to the lungs and release proteases (e.g., elastase), reactive oxygen species (ROS), and neutrophil extracellular traps (NETs) (11–13). These molecules exacerbate endothelial and epithelial damage, increasing vascular permeability and edema. Excessive neutrophil activation intensifies tissue injury, leading to alveolar collapse and hypoxemia (11, 13, 14). Resident lung macrophages initiate inflammation by releasing pro-inflammatory cytokines such as Tumor necrosis factor-α(TNF-α), Interleukin-1β (IL-1β), and Interleukin-6 (IL-6), as well as chemokines like Interleukin-8 (IL-8), which recruit neutrophils (15–18). These macrophages can polarize into pro-inflammatory (M1) or anti-inflammatory (M2) phenotypes (16–18). Circulating monocytes infiltrate the lungs, differentiating into macrophages that amplify cytokine storms and influence fibrotic responses. Dysregulated monocyte activation contributes to prolonged inflammation and fibrosis (13). Immune cells thus play dual roles in both the pathogenesis and resolution of ARDS, serving as key drivers of inflammation, therapeutic targets, and prognostic indicators.

Metabolic reprogramming plays a pivotal role in regulating immune cell subtypes. M1 macrophages rely on glycolysis and the pentose phosphate pathway (PPP) for rapid adenosine triphosphate (ATP) production and ROS generation (19). In contrast, M2 macrophages utilize oxidative phosphorylation (OXPHOS) and fatty acid oxidation (FAO) to promote tissue repair (20). Similarly, CD4+ T helper 1 (Th1) and Th17 cells depend on glycolysis and glutaminolysis for proliferation and cytokine production, including interferon-gamma (IFN-γ) and interleukin-17 (IL-17) (21). These examples demonstrate how specific metabolic pathways, such as glycolysis, OXPHOS, and FAO, are tailored to immune cell functions, offering potential therapeutic targets in cancer, autoimmunity, and infectious diseases. Metabolic reprogramming not only regulates immune cell subtypes but also improves energy metabolism balance and inhibits excessive inflammation. This process involves adaptive metabolic changes that cells undergo in response to alterations in their intra- and extracellular environment. Such adjustments allow cells to meet heightened demands for energy production and biosynthesis, essential for growth, proliferation, survival, and other cellular functions. This reprogramming encompasses several metabolic pathways, including glycolysis, OXPHOS, and lipid metabolism, thereby enhancing cellular resilience and function under challenging conditions (22, 23). In ARDS, various immune cells, such as alveolar macrophages, neutrophils, monocytes, and T lymphocytes, become activated (1). These cells release pro-inflammatory cytokines and chemokines, which amplify the inflammatory response and exacerbate tissue damage, particularly in conjunction with damaged epithelial and endothelial cells (3, 10). Investigating metabolic reprogramming can offer deeper insights into the metabolic regulation of immune cells under both physiological and pathological conditions, providing new therapeutic targets for disease treatment. For instance, targeting key metabolic pathways, such as glycolytic enzymes and lipid metabolism, may not only regulate immune cell phenotypes but also improve energy metabolism balance and inhibit excessive inflammatory responses, offering novel approaches for ARDS treatment (24–26). These results suggest that immune cell modulation and metabolic reprogramming are pivotal in the pathogenesis of ARDS. However, the exact mechanisms remain unclear and require further research.

**Figure 1:**
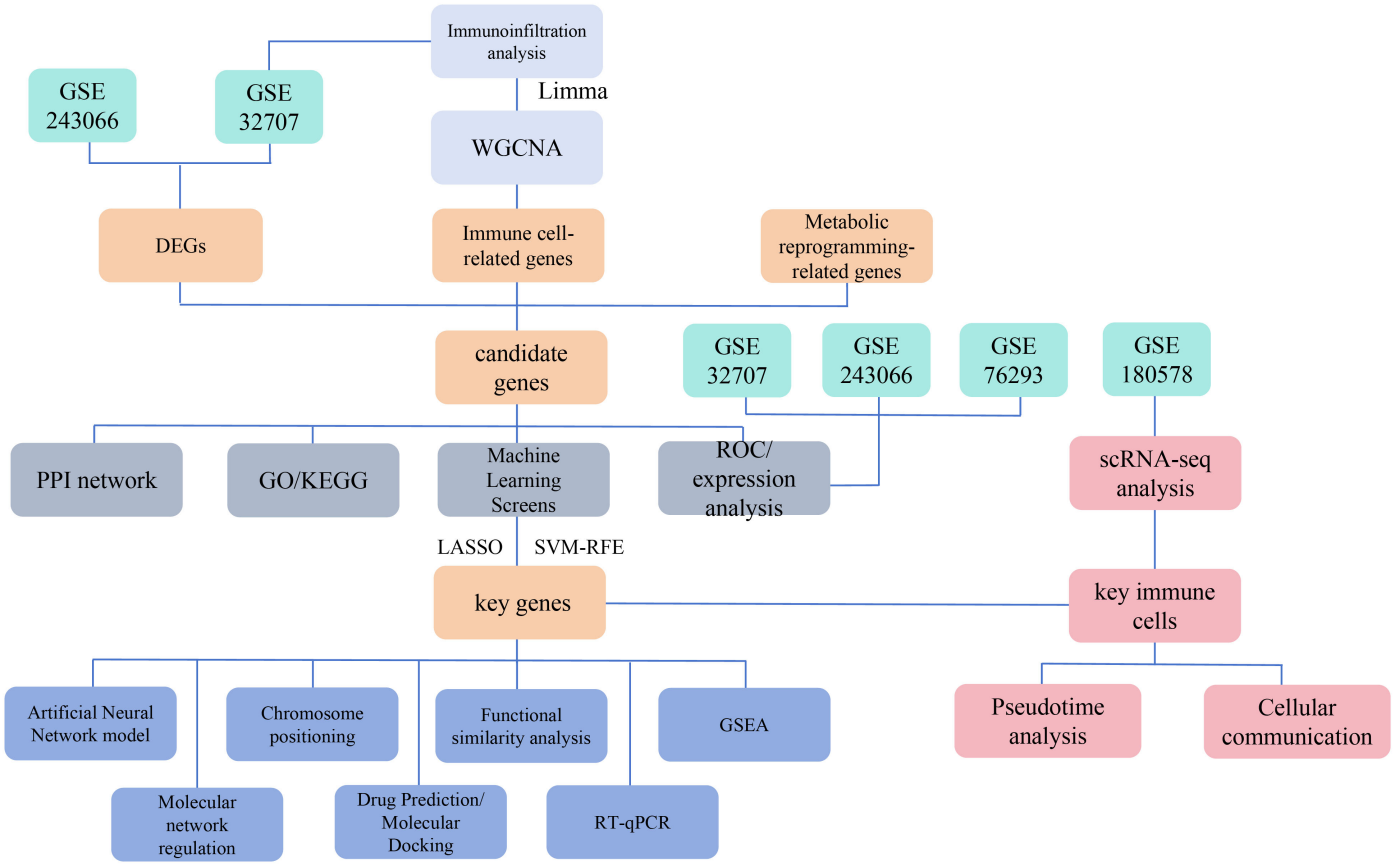
Schematic overview of the study design and workflow for identifying immune cell and metabolic reprogramming-related biomarkers in ARDS. This Figure depicts the multi-omic approach used to identify biomarkers associated with immune cells and metabolic reprogramming in acute respiratory distress syndrome (ARDS).

Given the limitations of current treatment strategies, identifying new biomarkers linked to immune cells and metabolic reprogramming is critically important. These biomarkers can enhance our understanding of ARDS pathogenesis and guide the development of targeted therapies. This study aims to identify biomarkers associated with immune cells and metabolic reprogramming in ARDS by integrating transcriptomic data, mechanistic research, and single-cell analysis (5–9). The study flowchart is shown in **Figure 1**. By examining the complex interplay between immune cell activity and metabolic changes in ARDS, this research has the potential to deepen our understanding of the syndrome and support the implementation of more effective clinical interventions.

## 2 Materials and methods

### 2.1 Data source

When selecting datasets related to ARDS with whole blood or peripheral blood mononuclear cell samples, the dataset should include a sufficient sample size and provide raw or processed gene expression data. Datasets with insufficient sample size or poor quality control will be excluded. ARDS-related datasets were obtained from the Gene Expression Omnibus (GEO) database (http://www.ncbi.nlm.nih.gov/geo/). The GSE32707 (GPL10558) dataset included 31 whole blood samples from patients with sepsis-induced ARDS and 34 whole blood samples from individuals without sepsis, systemic inflammatory response syndrome (SIRS), or ARDS (referred to as ARDS and control samples, respectively), which served as training set 1. The GSE243066 (GPL30209) dataset consisted of 34 whole blood samples from patients with ARDS and 15 healthy controls, serving as training set 2. The GSE76293 (GPL570) dataset included 12 blood polymorphonuclear neutrophil samples from patients with ARDS and 12 healthy controls, serving as the validation set. Additionally, the GSE180578 (GPL24676) dataset included single-cell RNA sequencing (scRNA-seq) data from peripheral blood mononuclear cells (PBMCs) collected from 4 patients with ARDS and 4 healthy controls. A total of 1,804 metabolism reprogramming-related genes (MRRGs) were retrieved from the literature (27) (**Supplementary Table 1**).

### 2.2 Detection of differentially expressed genes

DEGs1 between ARDS and control samples in training set 1 were identified using the limma (v 3.54.0) package (28), applying a threshold of |log_2_ fold-change (FC)| > 1 and P < 0.05. The signal intensity of chip data approximately follows a normal distribution. Limma, based on linear models and empirical Bayes methods, is specifically designed for such data and can effectively handle technical noise. For lowly expressed genes, Limma’s voom transformation enhances the detection ability of lowly expressed genes by applying weighted root mean square standard deviations. Similarly, DEGs2 between ARDS and control samples in training set 2 were identified using the Differential Expression Sequencing analysis (DESeq2, v1.42.0) package (29) with the same criteria. GSE243066 used a high-throughput sequencing platform, and the reads count of RNA-seq data followed a negative binomial distribution. DESeq2 directly models this distribution using a generalized linear model, avoiding information loss during the normalization process. DESeq2 uses the median ratio method for normalization, which is insensitive to extreme values and is suitable for handling outliers in sequencing data. Volcano plots for DEGs1 and heatmaps for DEGs2 were generated using the ggplot2 (v 3.5.1) package (30) and the ComplexHeatmap (v 2.14.0) package (31), respectively.

### 2.3 Immune infiltration analysis and weighted gene co-expression network analysis

To evaluate the infiltration of 64 immune cell types in training set 1, relative abundance was calculated using the xCell (v 1.1.0) package (32), and the proportional distribution of immune cells was visualized using ggplot2 (v 3.5.1). Differences in immune cell infiltration between ARDS and control samples in training set 1 were assessed using the Wilcoxon test, identifying immune cell types with significant differences in infiltration (P < 0.05), referred to as differential immune cells. Subsequently, WGCNA was performed on training set 1 using the WGCNA (v 1.71) package (33). Hierarchical clustering was applied to ARDS and control samples in training set 1 to identify and remove outlier samples. The optimal soft threshold (β) was selected when the scale-free fit index (R^2^) exceeded 0.9, and the average connectivity was near 0. The adjacency between genes was then calculated, with each module containing at least 50 genes. Co-expression modules were identified, and a hierarchical clustering tree was generated. Using differential immune cell scores as phenotypic features, the correlation between these scores and co-expression modules was calculated (|correlation coefficient (cor)| > 0.30, P < 0.05) (34). The co-expression modules with the highest positive and negative correlations with differential immune cell scores were selected as key modules, and the key module genes were subsequently identified.

### 2.4 Identification and functional analysis of candidate genes

The intersection of DEGs1, DEGs2, MRRGs, and key module genes was determined using the ggvenn (v 0.1.10) package (35), and the overlapping genes were designated as candidate genes. Subsequently, Gene Ontology (GO) and Kyoto Encyclopedia of Genes and Genomes (KEGG) enrichment analyses were performed on the candidate genes using the clusterProfiler (v 4.7.1.003) package (36, 37)(P < 0.05). The enrichment results from both GO and KEGG analyses were visualized with the GOplot (v 1.0.2) package (38). A protein-protein interaction (PPI) network (interaction score > 0.15) was constructed using the STRING database (https://www.string-db.org) and visualized using Cytoscape (v 3.7.1) software (39).

### 2.5 Identification of biomarkers

For candidate genes, Least Absolute Shrinkage and Selection Operator (LASSO) analysis was performed using the glmnet (v 4.1.4) package (40), with genes not penalized to zero selected for further analysis. Simultaneously, the caret (v 6.0-93) package (41) was employed to conduct Support Vector Machine Recursive Feature Elimination (SVM-RFE) analysis. The final feature genes were identified by intersecting the results from LASSO and SVM-RFE using the ggvenn (v 0.1.10) package. The expression patterns of these feature genes were then assessed in training set 1, training set 2, and the validation set (P < 0.05) to identify potential biomarkers. Finally, receiver operating characteristic (ROC) curves for these feature genes were plotted using the partial ROC (v 1.18.5) package (42), and genes with an area under the curve (AUC) > 0.7 were considered potential biomarkers across the three datasets.

### 2.6 Construction and evaluation of ANN model

To further validate the reliability of these biomarkers in predicting ARDS, In the GSE32707 dataset, key genes were selected as features, and all features were normalized using min-max scaling, adjusting the value range to (0, 1). Regarding the model architecture, the input layer consisted of 3 neurons, corresponding to the 3 feature genes, with a hidden layer containing 5 neurons and an output layer of 2 neurons for the binary classification task (ARDS and control). The activation function used the hyperbolic tangent function (tanh) for the hidden layer, and the Sigmoid function was applied to the output layer. The loss function chosen was cross-entropy (Cross Entropy), as it more effectively measures the difference between predicted and true labels in classification tasks. During training, the backpropagation algorithm was applied, with a learning threshold set at 0.1 and a random seed of 17 to ensure reproducibility. The optimization goal was to minimize the cross-entropy loss. Finally, the network structure was visualized using the plot() function from the neuralnet (v 1.44.2) package (43). ROC curves of the ANN model were then plotted for training set 1, training set 2, and the validation set, using the pROC (v 1.18.5) package, to assess the model’s performance (AUC > 0.7).

### 2.7 Chromosomal localization and enrichment analysis

The chromosomal localization of biomarkers was determined using the RCircos (v 1.2.2) package (44). Gene set enrichment analysis (GSEA) was performed on the biomarkers using the c2.cp.kegg.v7.4.symbols.gmt file obtained from the Molecular Signatures Database (MSigDB) (https://www.gsea-msigdb.org/gsea/msigdb/) as the background gene set. Spearman correlations between the biomarkers and other genes were calculated using the psych (v 2.2.9) package (45). Subsequently, GSEA was performed for each biomarker using the clusterProfiler (v 4.7.1.003) package, with the criteria |normalized enrichment score (NES)| > 1 and P < 0.05. The top 5 signaling pathways, ranked by descending P-value, were presented using the enrichplot (v 1.18.0) package (46). Additionally, genes associated with biomarker functions and their respective roles were predicted from the GeneMANIA database (http://genemania.org), and a gene-gene interaction (GGI) network was constructed.

### 2.8 Construction of regulatory networks

To predict miRNAs targeting the biomarkers, the DIANA-microT database (http://www.microrna.gr/microT) was used. The starBase database (https://rnasysu.com/encori/) was then employed to identify lncRNAs upstream of miRNAs (clipExpNum > 4), facilitating the construction of an lncRNA-miRNA-biomarker network. Transcription factors (TFs) targeting biomarkers were predicted using the NetworkAnalyst database (https://networkanalyst.ca/NetworkAnalyst/), resulting in the construction of a TF-biomarker network. Cytoscape (v 3.7.1) software was used to visualize these regulatory networks.

### 2.9 Drug prediction and molecular docking

The DrugBank database (https://go.drugbank.com/) was utilized to predict potential drugs targeting the biomarkers. The Cytoscape (v 3.7.1) software was again used to visualize the drug-biomarker network. Based on the highest-scoring drugs targeting the biomarkers, molecular docking was performed using the CB-Dock2 online tool (https://cadd.labshare.cn/cb-dock2/php/index.php) to assess the binding ability of the biomarkers to these drugs (binding energies ≤ -5 kcal/mol) (47). The three-dimensional structures of the drugs were obtained from the PubChem database (https://pubchem.ncbi.nlm.nih.gov/), and the biomarkers were imported into the Protein Data Bank (PDB) database (https://www.rcsb.org/) to retrieve their protein structures.

### 2.10 scRNA-seq analysis

For single-cell analysis, the GSE180578 dataset was processed using the Seurat (v 5.0.1) package (48). Cells and genes of low quality were filtered out (min.cells=3), and the remaining cells and genes were further selected based on stringent criteria (200 < nFeature_RNA < 3000, 200 < nCount_RNA < 15000, percent.mt < 20%). The filtered single-cell data were integrated using the Harmony function and normalized using the LogNormalize function. The top 2000 highly variable genes (HVGs) were identified using the vst method of the FindVariableFeatures function. Principal component analysis (PCA) was performed based on the HVGs, and a scree plot was generated using the Elbowplot function. The appropriate principal components (PCs) for downstream analysis were selected using the JackStraw function. Finally, high-quality cells were separated into distinct clusters using the FindNeighbors and FindClusters functions, with the uniform manifold approximation and projection (UMAP) clustering method. Marker genes for different clusters were identified using the FindAllMarkers function for further annotation (49–59) (**Supplementary Table 2**).

### 2.11 Cell communication analysis and identification of key cells

The ARDS and control sample data from the GSE180578 dataset were used to analyze cellular communication networks between different cell types using the CellChat (v 1.6.1) package (60). The interaction relationships of receptors and ligands for each cell type were determined. Additionally, the Wilcoxon test was applied to assess differences in the expression of biomarkers across different cell types in ARDS and control samples (P < 0.05) to identify key cells.

### 2.12 Pseudotime analysis

Key cells were selected from the GSE180578 dataset for further dimensionality reduction and clustering. The identified key cells were then reclustered and categorized into distinct cell subpopulations. Following this, to investigate the potential differentiation or activation trajectories of key cells during the pathogenesis of ARDS, cell pseudo-time trajectory analysis was conducted using the Monocle (v 2.30.0) package (61), and the cell differentiation trajectories were visualized using the DDRTree (v 0.1.5) package (62).

### 2.13 Reverse transcription-quantitative polymerase chain reaction

Twenty whole blood samples were collected from Zhongda Hospital of Southeast University, consisting of 10 samples from patients with ARDS and 10 from healthy controls. The study was approved by the Ethics Committee of Zhongda Hospital Southeast University (approval number: 2022ZDSYLL402-P01), and informed consent was obtained from all participants. Total RNA was extracted from the 20 samples using TRIzol reagent (Ambion, Cat#15596026, USA) following the manufacturer’s protocol. RNA concentration was measured using the NanoPhotometer N50. cDNA synthesis was carried out via reverse transcription using the SweScript RT II First Strand cDNA Synthesis Kit (Servicebio Cat#G3333,China), and the process was performed with the S1000TM Thermal Cycler (Bio-Rad, USA). Primer sequences for RT-qPCR are provided in **Supplementary Table 3**. RT-qPCR was performed using the CFX Connect Real-Time Quantitative Fluorescence PCR Instrument (Bio-Rad, USA), with β-actin used as the internal reference gene. The RT-qPCR results were analyzed using the 2-ΔΔCT method, exported to Excel, and then imported into GraphPad Prism 10.1.2 for statistical analysis and visualization (P < 0.05).

### 2.14 Experimental validation

#### 2.14.1 Animal experiment

C57BL/6 male mice (8–12 weeks old, 18–22 g) were purchased from Jiangsu Huachuang Xinnuo Pharmaceutical Technology Co., Ltd. (Taizhou, China) and were housed under specific pathogen-free conditions. All animal procedures were approved by the Institutional Animal Care and Use Committee of Southeast University (Approval No.SEU-20252018005).Mice were randomly divided into two groups: LPS group, mice were administered with LPS nasal instillation at a dose of 10 mg/kg(50ul); Control group, mice received an equal amount of saline. After 24 h, all mice were anesthetized and sacrificed. Blood samples were collected for subsequent neutrophils isolation. Lung tissues were harvested and divided into three portions: one was fixed in formalin for histological sectioning and HE staining, one was homogenized for cytokine analysis by Enzyme-Linked Immunosorbent Assay (ELISA), and the remainder was stored at -80°C for RT-qPCR analysis.RPL14, SMARCD3, and TCN1 mRNA expression in peripheral blood neutrophils and lung tissues was analyzed by RT-qPCR. Levels of glutathione (GSH), IL-6, and TNF-α in lung tissue homogenates were measured by ELISA.

#### 2.14.2 Hematoxylin and eosin staining

Tissue samples were paraffin-embedded and sectioned (4-5 μm). After deparaffinization by eco-friendly deparaffinisation solution (Servicebio, Cat #G1128,China), 20 min×2,graded ethanol(Sinopharm Chemical Reagent Co., Ltd., Cat #100092683, China), 100%-75%, 5 min×3) and rinsing. H&E staining was performed by an HE HD constant dye kit according to the manufacturer’s protocol (Servicebio, Cat #G1076, China). Stained sections were examined under brightfield microscopy (Nikon Eclipse C1).

#### 2.14.3 Isolation of neutrophils from mouse peripheral blood

As described in previous studies, neutrophils were collected from the whole blood of C57BL/6 mice by using a mouse peripheral blood neutrophil separator kit (Solarbio, Cat# P9201, China) according to the instructions (64, 65). Mice were administered with LPS nasal instillation to activate neutrophils. 24 hours later, blood was collected via cardiac puncture into anticoagulant tubes. Within two hours, blood was diluted 1:1 and layered onto a separation medium, followed by centrifugation (1500 × g, 30 min, 25°C). Neutrophils were collected from the interface, lysed to remove RBCs, washed with PBS (pH 7.4), and either stored at -80°C or used immediately.

#### 2.14.4 Cell culture and transfection

Tohoku Hospital Pediatrics-1(THP-1)cells were originally obtained from Guangzhou Cellcook Biotech Co., Ltd. The cells were maintained in RPMI 1640 medium (Gibco, Cat#11875093,USA) supplemented with 10% heat-inactivated fetal bovine serum (FBS) and 1% penicillin-streptomycin (Beyotime, Cat#ST488S,China) at 37°C in a humidified 5% CO_2_ atmosphere. For differentiation, THP-1 cells were treated with 100 ng/ml phorbol 12-myristate 13-acetate (PMA) (Macklin, Cat#C708929, China) (63). After 24-hour treatment with PMA to differentiate THP-1 cells into macrophages, the cells were subsequently stimulated with 1 μg/mL lipopolysaccharide (LPS) (Beyotime, Cat# ST1470, China).

RPL14 siRNA, SMARCD3 siRNA, TCN1 siRNA, and negative control siRNA were synthesized by GENCEFE Biotech Co., Ltd. (Wuxi, China) siRNA sequences are provided in **Supplementary Table 4**.The siRNAs were reverse-transfected into cells using Lipofectamine 2000 (Invitrogen, Cat#11668019,USA) according to the manufacturer’s protocol. After 24 hours of transfection to allow for gene silencing, the cells were stimulated with 1 μg/ml LPS.

#### 2.14.5 ROS detection

Cells were incubated with 2’,7’-dichlorodihydrofluorescein diacetate (DCFH-DA) from the ROS Assay Kit (Beyotime, Cat# S0033S,China) for 20 min at 37°C under light-protected conditions. Excess dye was removed by washing with PBS. Fluorescence intensity was measured using fluorescence microscope with excitation wavelengths of 485/530 nm, where increased signal intensity correlated with elevated intracellular ROS levels. Untreated and H_2_O_2_-treated cells were included as controls.

#### 2.14.6 JC-1 staining for mitochondrial membrane potential analysis

Cells were incubated with JC-1 dye (Beyotime, Cat #C2003S, China) for 30 min at 37°C in the dark. After incubation, excess dye was removed by washing twice with phosphate-buffered saline (PBS). Mitochondrial membrane potential was assessed by fluorescence microscopy (Olympus CKX53).

#### 2.14.7 Tunel staining assay

Fixed samples were permeabilized with 0.1% Triton X-100 (Beyotime, Cat# ST1723,China) for 5 min and then incubated with TUNEL reaction mix from the Tunel staining Assay Kit(Beyotime, Cat #C1088, China) for 60 min at 37°C protected from light. The samples were washed and counterstained with DAPI. Apoptotic cells (TUNEL-positive) were quantified by fluorescence microscopy.

#### 2.14.8 ELISA

AN assay for the determination of TNF-α,IL-6, GSH from THP-1 cells with SiRNA transfection and homogenate of mouse lung tissue had been validated using a commercially available enzyme-linked immunosorbent assay (ELISA) kit(Human TNF-α High Sensitivity ELISA Kit, MULTI SCIENCE, Cat# EK182HS-AW1, China; Human IL-6 ELISA Kit, Solarbio, Cat#SEKM-0013, China;Mouse IL-6 ELISA Kit, Solarbio, Cat#SEKM-0007, China; Total Glutathione Assay Kit, Beyotime, Cat#S0052, China; Mouse TNF-α ELISA Kit, Solarbio, Cat# SEKM-0034,China)following manufacturer’s protocol.

#### 2.14.9 Lactate content assay

THP-1 cell supernatant was collected after siRNA transfection and LPS treatment. Lactate concentration was measured using a WST-8-based kit(Beyotime, Cat # S0208S, China). Samples were incubated with the reaction mixture at 37°C for 30 min, and absorbance was read at 450 nm. Results were calculated via a lactate standard curve.

#### 2.14.10 Glucose uptake assay

Glucose uptake assay was measured using a WST-8-based kit(Beyotime, Cat # S0554, China).After siRNA and LPS treatment, THP-1 cells were glucose-starved, incubated with 2-DG, and lysed. Lysates were reacted with a WST-8 working solution, incubated at 37°C for 30 min, and absorbance was measured at 450 nm. Glucose uptake was quantified using a 2-DG6P standard curve.

#### 2.14.11 Glucose consumption assay

Following THP-1 cells treatment with siRNA and LPS, culture supernatants are collected after a defined incubation period and centrifuged to remove debris. The glucose concentration in the supernatant is quantified using a glucose oxidase-based assay kit(Beyotime, Cat # S0202M, China) by measuring absorbance at 490–540 nm. Glucose consumption is calculated by subtracting the glucose concentration at the end of the experiment from the initial concentration, normalized to total protein or cell number to assess cellular metabolic activity.

#### 2.14.12 Cyclosporine A drug treatment

Induction of THP-1 cell differentiation into macrophages via PMA treatment and then a macrophage inflammatory model was constructed by LPS. Different concentrations of CsA were applied to the macrophages, which was divided into three groups: low concentration group (2μM), medium concentration group (4μM) and high concentration group(8μM). RT-qPCR was performed to measure the mRNA expression levels of IL-6 and TNF-α in each group. RPL14,SMARCD3 and TCN1 mRNA expression also was detected by RT-qPCR. Cells from the above groups were further assessed for mitochondrial function by JC-1 staining, oxidative stress by ROS detection, apoptosis by TUNEL staining assay and inflammatory markers (TNF-α, IL-6, GSH) via ELISA.

### 2.15 Statistical analysis

Bioinformatics analyses were performed using R (v 4.2.2). This study first identified differentially expressed genes (DEGs1) in training set 1 using the limma package (v 3.54.0) with the criterion of |log2fold-change (FC)| > 1 and P < 0.05. Using the same threshold criteria, DEGs2 were identified in training set 2 with the DEseq2 package (v 1.42.0). Based on these candidate genes, LASSO regression analysis was performed using the glmnet package (v 4.1.4) in R software, with the family parameter set to binomial and ten-fold cross-validation to select the feature genes. Subsequently, the caret package (v 6.0-93) in R was used to further screen feature genes using the random forest method, and recursive feature elimination (RFE) was applied to iteratively remove unimportant genes, ultimately obtaining the selected feature genes. Next, the Wilcoxon test was used to compare the expression differences of key genes between ARDS and control samples in the main training set (GSE32707), auxiliary training set (GSE243066), and validation set (GSE76293), with FDR correction applied for multiple testing and a significance threshold of p < 0.05. Additionally, the Wilcoxon test was used to analyze immune cell infiltration differences between disease and normal sample groups, with the Benjamini-Hochberg method for multiple testing correction (α=0.05).

## 3 Results

### 3.1 Acquisition of differentially expressed genes and module genes

**Figure 2:**
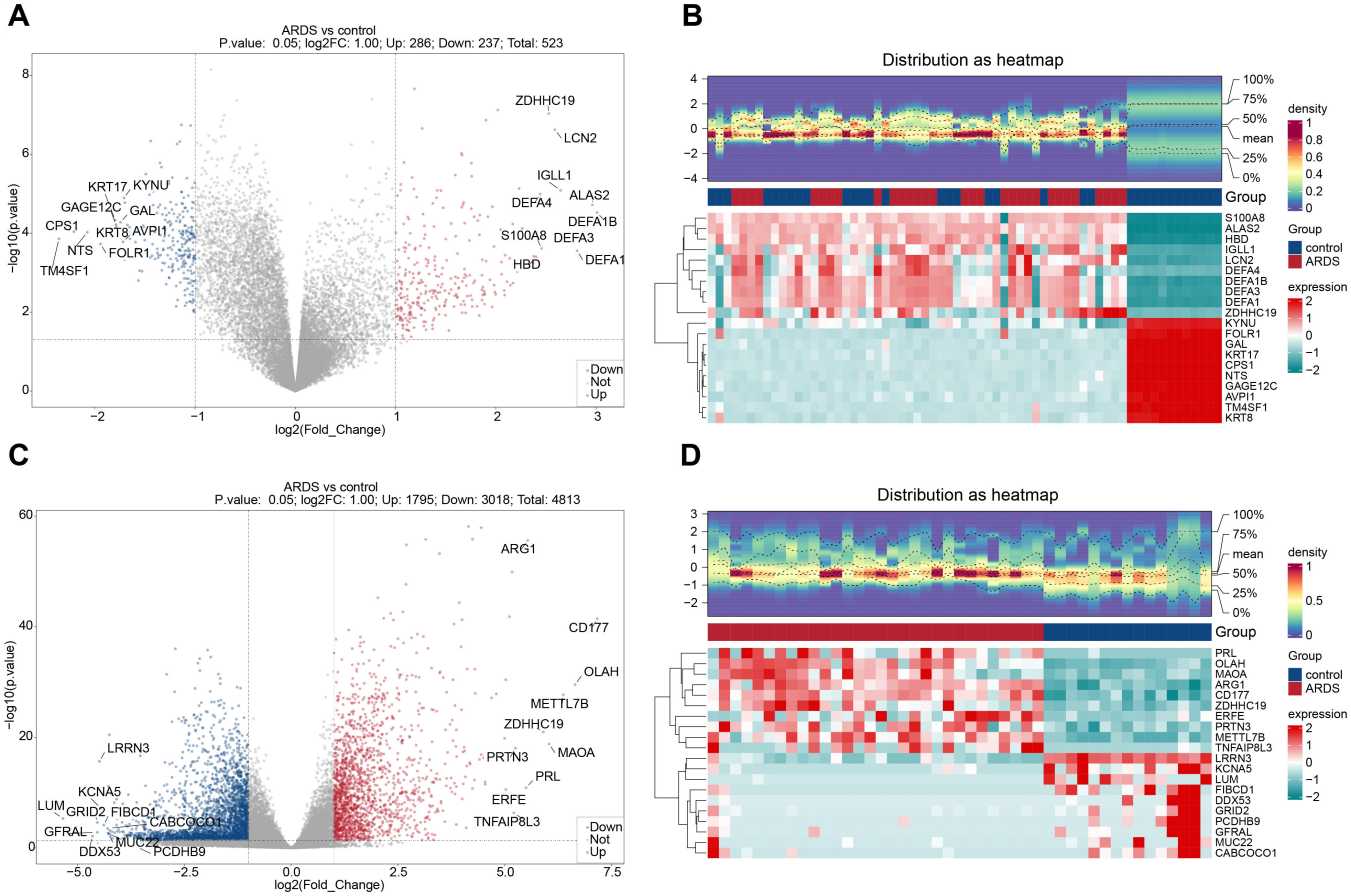
Identification of differentially expressed genes (DEGs) in ARDS. **(A)** Volcano plot of DEGs in training set 1 (GSE32707). **(B)** Heatmap of DEGs in training set 1. **(C)** Volcano plot of DEGs in training set 2 (GSE243066). **(D)** Heatmap of DEGs in training set 2.

**Figure 3:**
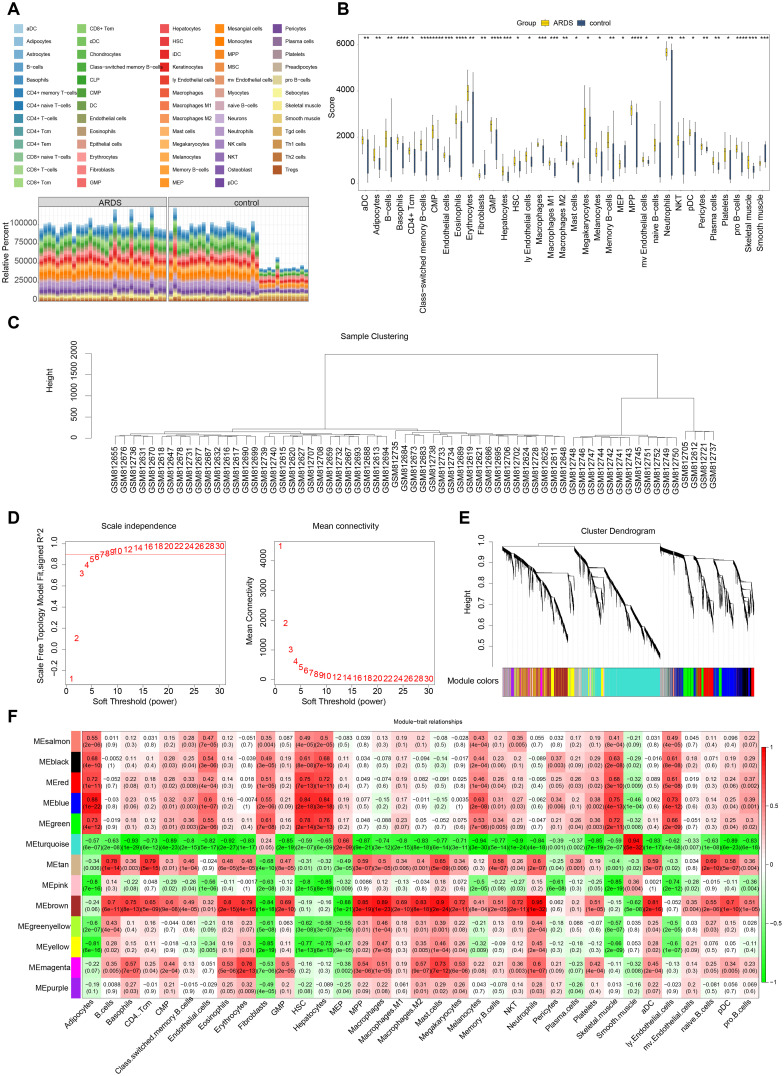
Immune infiltration analysis and weighted gene co-expression network analysis (WGCNA). **(A)** Analysis of immune cell infiltration. **(B)** Differential immune cell infiltration analysis. **(C)** Sample clustering and detection of outliers. **(D)** Determination of the optimal soft threshold (β). **(E)** Cluster dendrogram of genes, with different colors below representing different co - expression modules. **(F)** Heatmap displaying the correlation between gene modules (rows) and cell type proportions (columns), where color intensity indicates the strength of correlation.

A total of 523 dDEGs1 were identified in training set 1 through differential expression analysis, with 286 up-regulated and 237 down-regulated genes. In training set 2, 4,813 DEGs2 were detected, including 1,795 up-regulated and 3,018 down-regulated genes. The top 10 up-regulated and down-regulated DEGs and their expression profiles were displayed on volcano plots and heatmaps, respectively (**Figures 2A-D**). In both ARDS and control samples, the infiltration levels of 64 immune cell types were presented in a stacked plot. **Supplementary Table 5** displays the top 10 immune cells with the highest infiltration abundance, with neutrophils exhibiting the most significant infiltration (**Figure 3A**, **Supplementary Table 5**). It is worth noting that some of the control samples exhibited a relatively high proportion of immune cells. This phenomenon may have reflected individual differences during sample collection, such as potential subclinical infections, inflammatory responses, or other undefined physiological states, and these factors may have caused the non-specific activation and recruitment of immune cells in the control group. A total of 35 immune cells showed significant differences in infiltration (**Figure 3B**, **Supplementary Table 6**). Following this, WGCNA was conducted using differential immune cell scores as traits. No abnormal samples were found in training set 1 (**Figure 3C**). The β value was determined to be 9 (**Figure 3D**). A co-expression matrix was then constructed, and 14 gene modules were identified (**Figure 3E**). Among these, MEbrown (cor=0.95) and MEturquoise (cor=-0.94), which showed the largest positive and negative correlations with differential immune cell scores, respectively, were considered as key modules (**Figure 3F**). These modules contained a total of 6,601 key module genes.

### 3.2 27 candidate genes were screened out and were significantly enriched in ribosomal function and amino acid metabolic pathways

**Figure 4:**
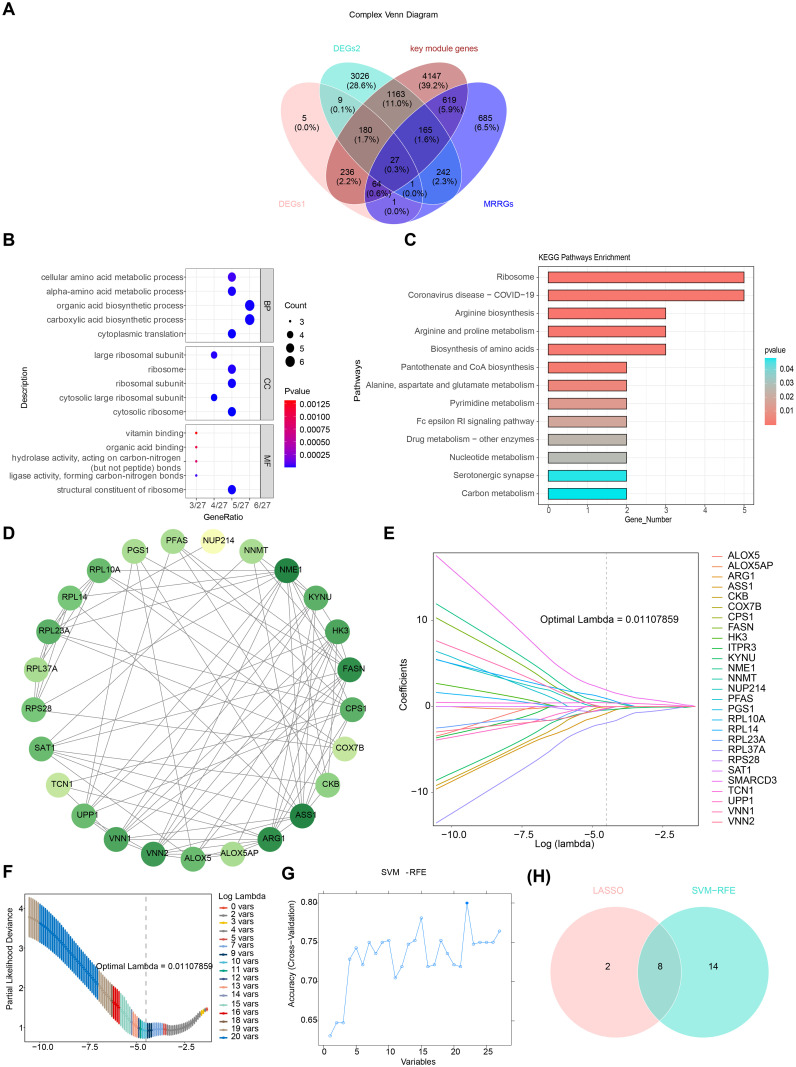
Identification and validation of potential biomarkers in ARDS. **(A)** Venn diagram of overlapping genes. **(B)** Gene Ontology (GO) enrichment analysis of 27 candidate genes, highlighting significant enrichment in biological processes such as “structural constituent of ribosome.” **(C)** Kyoto Encyclopedia of Genes and Genomes (KEGG) enrichment analysis, focusing on pathways related to amino acid biosynthesis and metabolism. **(D)** Protein-protein interaction (PPI) network of candidate genes, with key genes such as NME1 and ASS1 showing high connectivity. **(E)** LASSO coefficient profiles for the 10 selected feature genes. **(F)** Cross-validation for tuning parameter selection in LASSO analysis. **(G)** SVM-RFE analysis for feature selection. **(H)** Venn diagram illustrating the overlap of feature genes identified by LASSO and SVM-RFE.

After overlapping DEGs1, DEGs2, key module genes, and MRRGs, 27 candidate genes were identified (**Figure 4A**). Enrichment analysis revealed that the 27 candidate genes were significantly associated with 408 GO terms, including “structural constituent of ribosome” (**Figure 4B**, **Supplementary Table 7**). Additionally, KEGG analysis identified 101 enriched terms, primarily linked to amino acid biosynthesis and metabolic pathways, such as those involving arginine, proline, and glutamate (**Figure 4C**, **Supplementary Table 8**). These results suggest a strong association between the candidate genes and ribosomal function, as well as amino acid metabolism. In the PPI network, two discrete targets were removed, and genes like NME1 and ASS1 showed strong connectivity with other genes, highlighting their potential relevance (**Figure 4D**).

### 3.3 RPL14, SMARCD3, and TCN1 were identified as potential biomarkers of ARDS

Through LASSO analysis, 10 feature genes were selected, including RPL37A, VNN2, RPS28, NME1, TCN1, RPL14, SMARCD3, ASS1, KYNU, and CKB (**Figures 4E, F**). The SVM-RFE analysis identified 22 feature genes, including SMARCD3, TCN1, UPP1, RPL37A, HK3, KYNU, VNN1, NUP214, RPL14, PGS1, RPS28, RPL10A, ARG1, RPL23A, PFAS, CKB, ITPR3, ASS1, ALOX5, ALOX5AP, SAT1, and FASN (**Figure 4G**). A final set of 8 feature genes was derived (**Figure 4H**).

**Figure 5:**
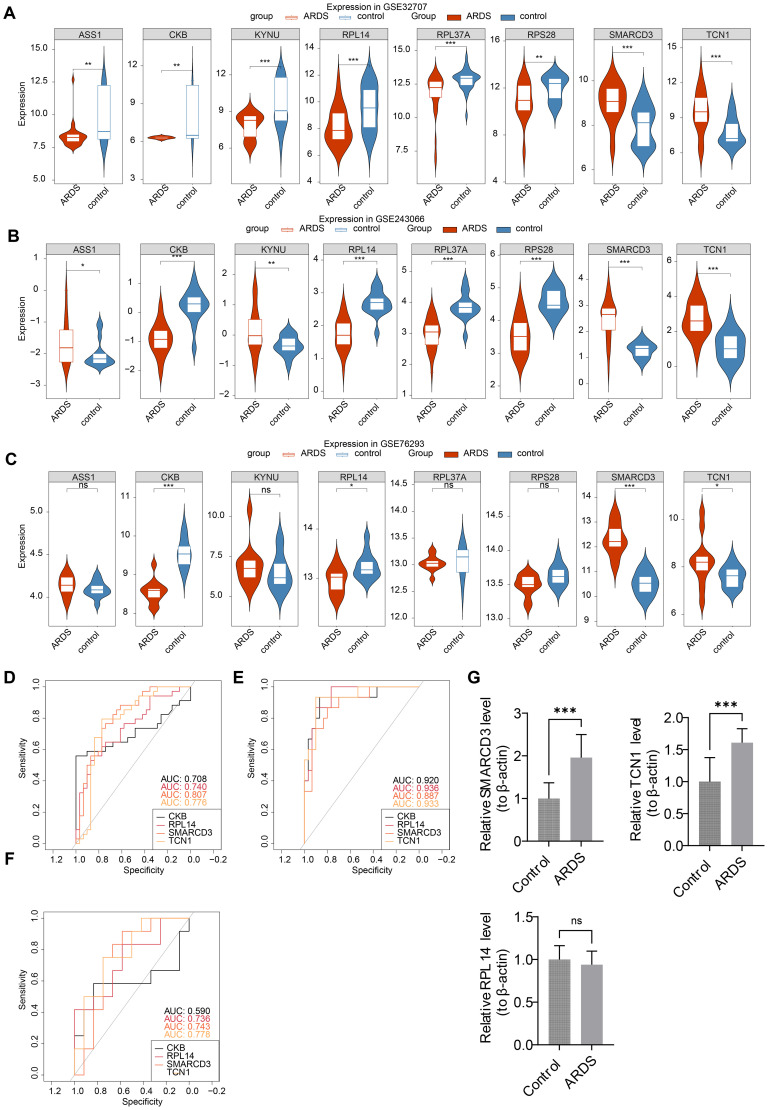
Identification and validation of biomarkers in ARDS. **(A-C)** Expression analysis of candidate biomarkers. **(D-F)** ROC curve analysis for the performance of biomarkers. **(G)** RT-qPCR validation of biomarkers. *p<0.05, **p<0.01, ***p<0.001 vs control group. "ns" indicates no significance.

Expression analysis indicated that CKB and RPL14 were significantly down-regulated in ARDS samples across training set 1, training set 2, and the validation set. Conversely, SMARCD3 and TCN1 were up-regulated in ARDS samples across all datasets. The expression patterns of ASS1 and KYNU were inconsistent, and no significant expression differences for RPL37A and RPS28 were observed (**Figures 5A-C**). Consequently, CKB, RPL14, SMARCD3, and TCN1 were identified as candidate biomarkers. Furthermore, the AUC values for RPL14, SMARCD3, and TCN1 exceeded 0.7 across the three datasets (**Figures 5D-F**), supporting their potential as reliable biomarkers.

### 3.4 SMARCD3 and TCN1 were highly expressed in ARDS samples

To further validate these biomarkers, RT-qPCR analysis was performed using human whole-blood samples. The results confirmed a significant upregulation of SMARCD3 and TCN1 in ARDS samples (P < 0.05) (**Figure 5G**), consistent with the findings from training set 1, training set 2, and the validation set. While RPL14 showed a downward trend in ARDS samples, the difference was not statistically significant (P=0.44).

### 3.5 The ANN model of biomarkers was constructed and evaluated

**Figure 6:**
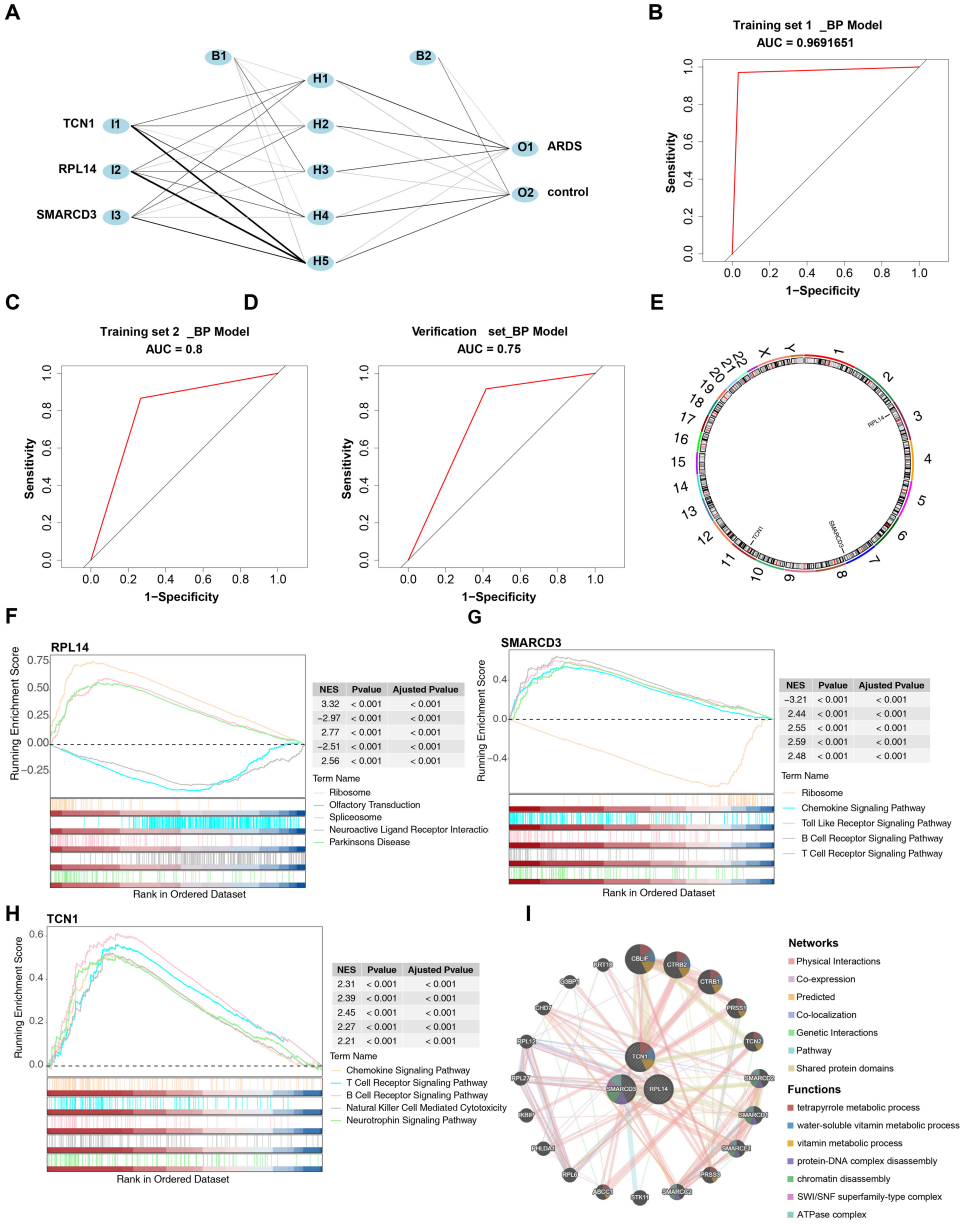
Construction and evaluation of the ANN model and functional enrichment analysis of biomarkers. **(A)** Architecture of the ANN model. **(B-D)** ROC curve analysis for the ANN model. **(E)** Chromosomal localization of biomarkers. **(F-H)** GSEA of biomarkers. **(I)** Gene-gene interaction (GGI) network of biomarkers.

Based on these biomarkers, an ANN model was constructed to evaluate their predictive power in ARDS onset (**Figure 6A**). The ROC curve analysis showed that the AUC values for training set 1, training set 2, and the validation set were 0.97 (specificity: 0.9706, sensitivity: 0.9677), 0.80 (specificity: 0.8670, sensitivity: 0.7330), and 0.75 (specificity: 0.9170, sensitivity: 0.5830), respectively (**Figures 6B-D**).

### 3.6 The core biomarkers were found to be involved in the pathogenesis of ARDS by regulating immune signaling and ribosomal pathways

RPL14, SMARCD3, and TCN1 were mapped to chromosomes 3, 7, and 7, respectively (**Figure 6E**), suggesting that these three biomarkers may have distinct biological functions. GSEA revealed that RPL14, SMARCD3, and TCN1 were enriched in 82, 89, and 69 pathways, respectively (**Supplementary Tables 9**–**11**). Notably, the ribosome pathway was significantly associated with both RPL14 and SMARCD3, while chemokine, B cell receptor, and T cell receptor signaling pathways were uniquely linked to both SMARCD3 and TCN1 (**Figures 6F-H**). These findings indicate that these biomarkers may contribute to ARDS by modulating T/B cell immune responses, in addition to the inflammatory response.

The GGI network constructed revealed the top 20 genes associated with the function of the biomarkers, such as CBLIF, which are involved in water-soluble vitamin metabolic processes (**Figure 6I**).

### 3.7 The regulatory networks of RPL14, SMARCD3, and TCN1 were constructed

**Figure 7:**
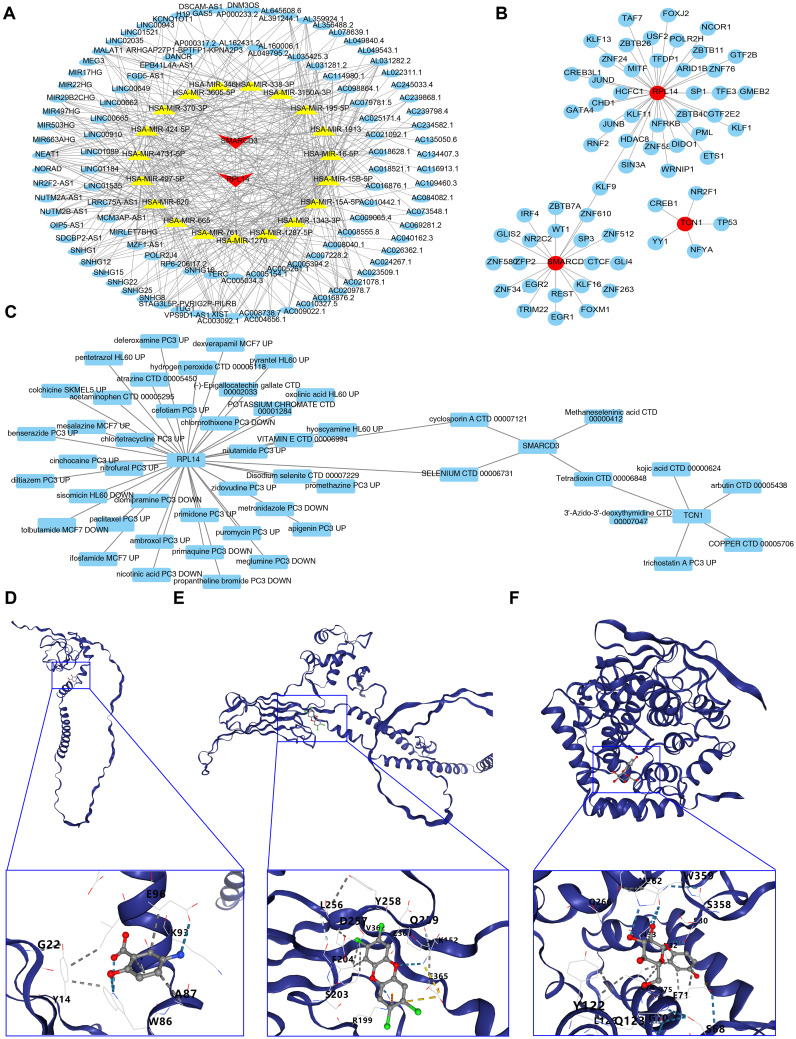
Regulatory networks, drug prediction and molecular docking analysis for biomarkers. **(A, B)** Regulatory networks associated with biomarkers. **(C)** Drug-biomarker interaction network. **(D-F)** Molecular docking analysis of drugs targeting biomarkers.

A total of 44 miRNAs were predicted to target RPL14 and SMARCD3, while no miRNA targeting TCN1 was identified. Based on a threshold of clipExpNum > 4, 107 lncRNAs were predicted by the 20 miRNAs. Consequently, a lncRNA-miRNA-mRNA network involving 2 biomarkers, 20 miRNAs, and 107 lncRNAs was constructed (**Figure 7A**). Additionally, 62 TFs targeting RPL14, SMARCD3, and TCN1 were identified, and the TF-biomarker network was generated (**Figure 7B**). Among these, KLF9 was found to target both RPL14 and SMARCD3, suggesting common transcriptional regulation in ARDS for these biomarkers.

### 3.8 Selenium and cyclosporin A were screened as potential drugs and molecular docking was conducted

The drug-biomarker network revealed that 41 drugs targeted RPL14, 4 targeted SMARCD3, and 6 targeted TCN1. Notably, tetradioxin (CTD 00006848) was identified as a drug targeting both SMARCD3 and TCN1, while selenium (CTD 00006731) and CsA (CTD 00007121) were found to target both RPL14 and SMARCD3 (**Figure 7C**, **Supplementary Table 12**). Molecular docking was then performed using the highest-scoring drugs targeting the biomarkers. Mesalazine (MCF7 UP) and arbutin (CTD 00005438) showed the highest scores for RPL14 and TCN1, respectively. For SMARCD3, although selenium (CTD 00006731) and methaneseleninic acid (CTD 00000412) had relatively high scores, their 3D structures were unavailable, so tetradioxin (CTD 00006848) was used instead. The binding energies of the complexes formed by RPL14 and mesalazine (**Figure 7D**), SMARCD3 and tetradioxin (**Figure 7E**), and TCN1 and arbutin (**Figure 7F**) were -4.5, -6.8, and -7.1 kcal/mol respectively, with the binding centers located at (-4, -3, -1), (-22, 38, 4), and (-15, -3, 10) (**Supplementary Table 13**). The binding energy of RPL14 and mesalazine did not reach -5 kcal/mol, indicating a relatively low affinity between the ligand and receptor, suggesting that the complex formed might be easily dissociated. Overall, these results highlight the potential therapeutic relevance of RPL14, SMARCD3, and TCN1, particularly in the context of ARDS treatment.

### 3.9 Annotation yielded 8 cell types

**Figure 8:**
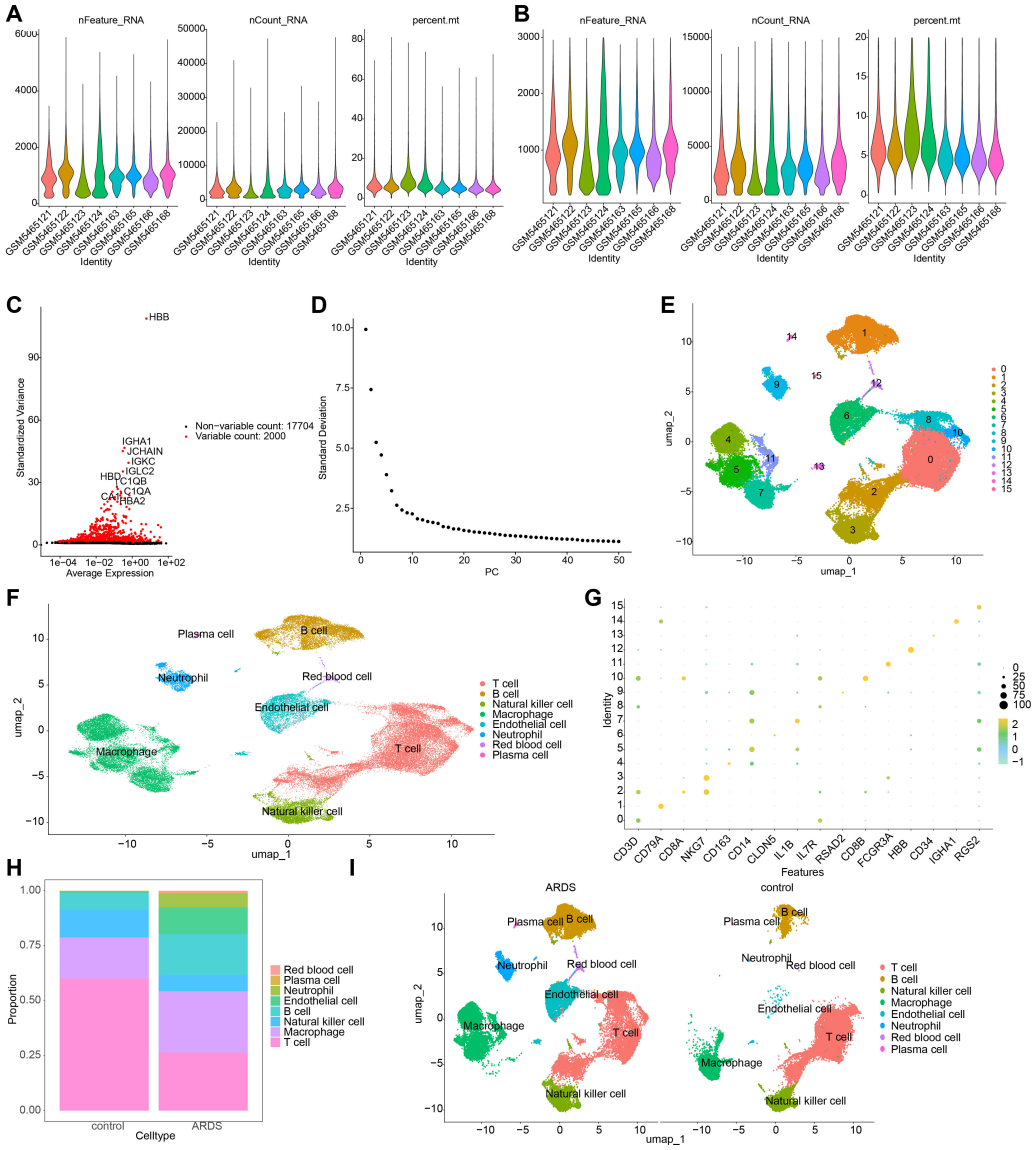
Single-cell RNA sequencing (scRNA-seq) analysis of ARDS samples. **(A, B)** Number of cells and genes before and after quality control. **(C)** UMAP plot depicting the top 2,000 highly variable genes (HVGs). **(D)** Scree plot for principal component analysis (PCA), with the top 30 principal components selected for further analysis. **(E)** UMAP plot showing the identification of 16 distinct cell clusters. **(F)** Annotation of 8 major cell types, including B cells, macrophages, and neutrophils. **(G)** Expression patterns of marker genes across different cell types. **(H)** Bar plot depicting the proportion of cell types in ARDS and control samples. **(I)** UMAP plot emphasizing the enrichment of macrophages, endothelial cells, and neutrophils in ARDS samples.

In the GSE180578 dataset, prior to quality control, a total of 57,811 cells and 19,704 genes were identified. After quality control, 53,816 cells and 19,704 genes were retained (**Figures 8A, B**). The top 2,000 HVGs and the top 30 PCs were then used for UMAP clustering (**Figures 8C, D**). This process led to the classification of all high-quality cells into 16 distinct clusters (**Figure 8E**). The cell clusters were annotated, revealing 8 cell types, including B cells, endothelial cells, macrophages, natural killer cells, neutrophils, plasma cells, red blood cells, and T cells (**Figure 8F**). The marker genes displayed high specificity across different cell clusters (**Figure 8G**). Notably, T cells comprised the highest proportion among all cell types. A comparison between ARDS and control samples revealed that macrophages, endothelial cells, and neutrophils were significantly more abundant in ARDS samples (**Figures 8H, I**).

### 3.10 Macrophages and neutrophils were recognized as key cells in ARDS

**Figure 9:**
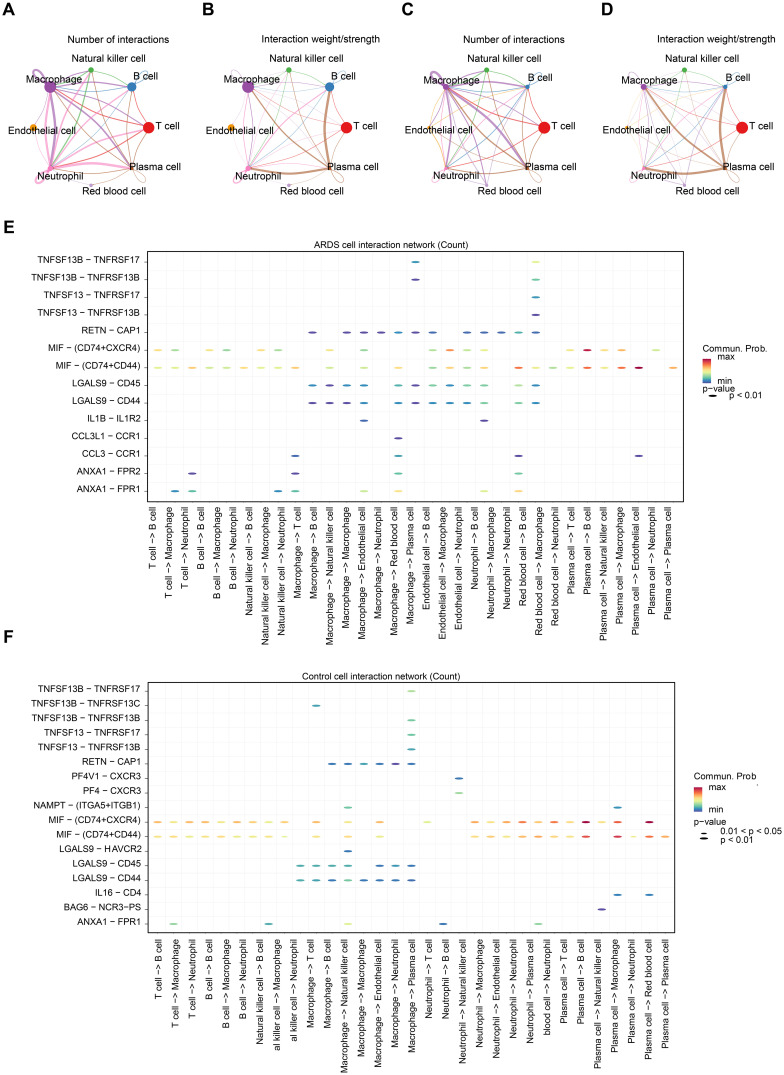
Cell-cell communication and key cell identification in ARDS. **(A)** The cell communication network count plot between ARDS samples. The thickness of the lines represents the quantity of interactions between cells. **(B)** The cell communication network weight plot between ARDS samples. The thickness of the lines represents the strength of interactions between cells. **(C)** The cell communication network count plot between control samples. **(D)** The cell communication network weight plot between control samples. **(E)** Bubble plot of receptor ligand interactions in the ARDS samples. The y-axis represented different receptor-ligand pairs, and the x-axis represented different cell-cell interactions. The color of the legend on the right ranged from blue to red, with the redder the color, the stronger the interaction. **(F)** Bubble plot of receptor ligand interactions in control sample.

**Figure 10:**
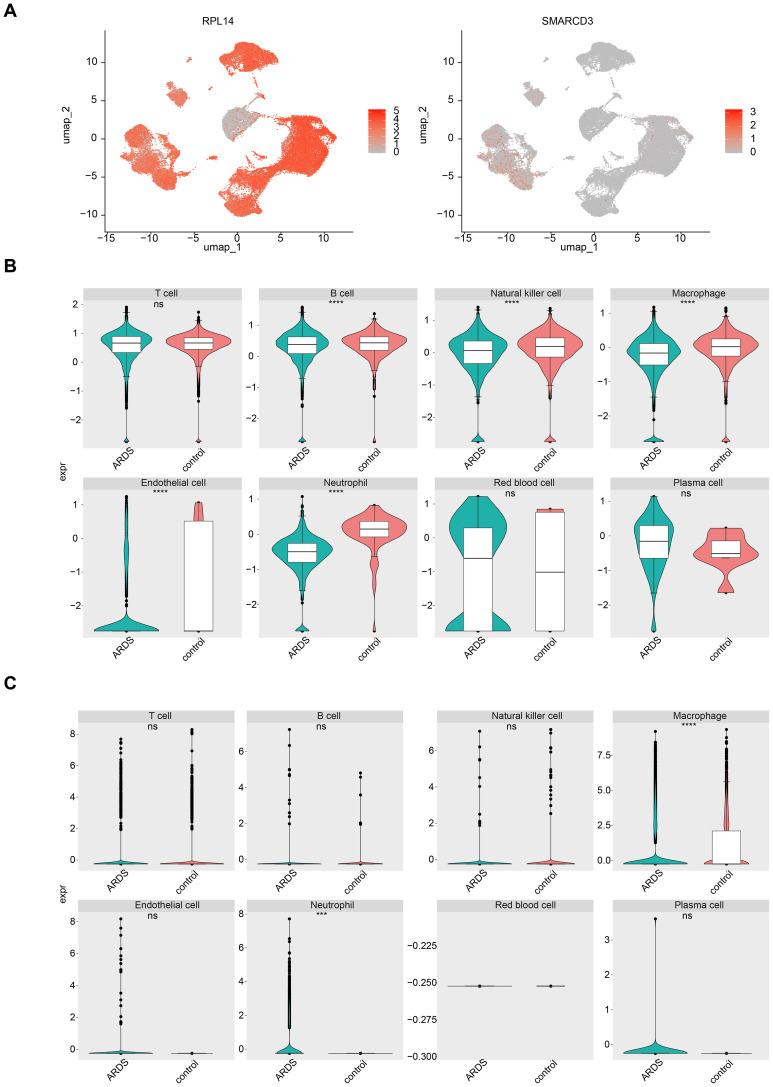
The expression of RPL14 (H) and SMARCD3 in cells. **(A)** UMAP plot showing the expression of RPL14 and SMARCD3 in different cell types. **(B, C)** Box plots illustrating significant differential expression of RPL14 and SMARCD3 in macrophages and neutrophils. ***p<0.001,****p<0.0001,vs control group. "ns" indicates no significance.

The cell-cell communication network analysis showed that, in ARDS samples, macrophages and neutrophils exhibited a greater number of interactions with other cell types compared to control samples (**Figures 9A, C**). This suggests that macrophages and neutrophils may play more pivotal roles in ARDS than other cell types. In ARDS samples, the interaction intensity of Neutrophils with other cells was stronger than that in control samples (**Figures 9B, D**). **Figures 9E, F** display the receptor-ligand pairings between different cell types in ARDS and control samples. Among these pairings, MIF-(CD74+CXCR4) and MIF-(CD74+CD44) were the core regulatory pathways for intercellular communication in both types of samples, and they could collectively regulate a variety of intercellular interactions, including T cell→Macrophage, Plasma cell→B cell, and Plasma cell→Macrophage. Furthermore, the expression patterns of RPL14, SMARCD3, and TCN1 at the cellular level were examined. It was found that RPL14 and SMARCD3 were expressed in multiple cell types, whereas TCN1 was not expressed (**Figure 10A**). Among these, RPL14 and SMARCD3 showed significant differential expression in macrophages and neutrophils (**Figures 10B, C**), making them key cells for further investigation.

### 3.11 RPL14 and SMARCD3 expression changes during the differentiation of key cells were investigated

**Figure 11:**
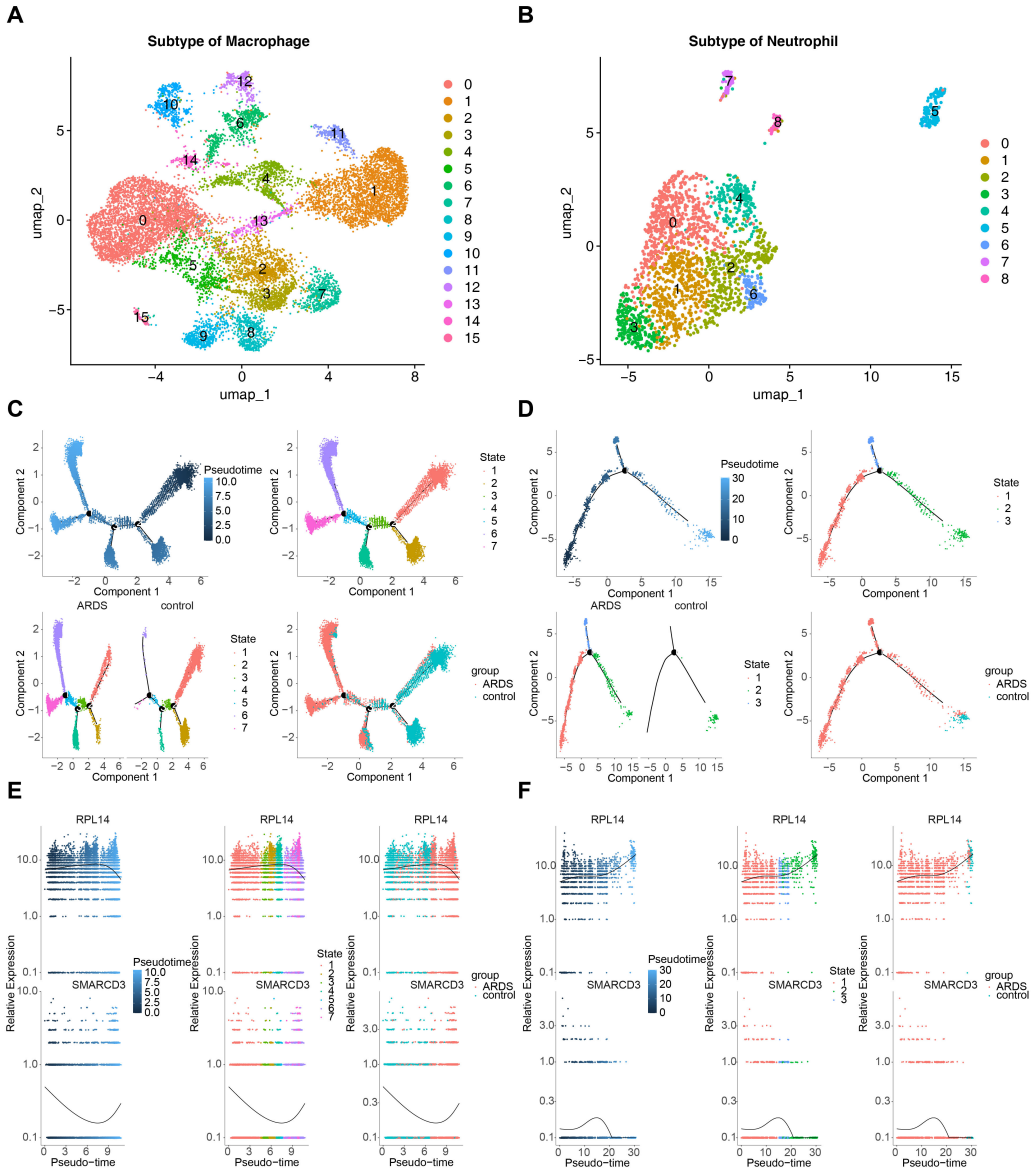
Pseudotime analysis of key cells in ARDS. **(A)** UMAP plot showing 16 subtypes of macrophages. **(B)** UMAP plot showing 9 subtypes of neutrophils. **(C)** Pseudotime trajectory of macrophages with 7 differentiation states. **(D)** Pseudotime trajectory of neutrophils with 3 differentiation states. **(E)** Expression trends of RPL14 and SMARCD3 during macrophage differentiation. **(F)** Expression trends of RPL14 and SMARCD3 during neutrophil differentiation.

Secondary dimensionality reduction clustering analysis was performed on the key cells. It was observed that macrophages were divided into 16 subtypes, and neutrophils were divided into 9 subtypes (**Figures 11A, B**). These subtypes were then placed along a differentiation trajectory based on differentiation time sequence, with darker shades of blue corresponding to earlier stages of differentiation. Macrophages were found to have 7 differentiation states, with State 1 representing the earliest and most specific stage (**Figure 11C**). Neutrophils exhibited 3 differentiation states, with State 1 also being the earliest and most specific (**Figure 11D**).

As macrophages differentiated, the expression of RPL14 increased initially and then decreased, while the expression of SMARCD3 followed an opposite trend (**Figure 11E**). In neutrophils, RPL14 expression generally trended upward, whereas SMARCD3 expression initially increased and then decreased (**Figure 11F**). These results suggest that the expression trends of RPL14 and SMARCD3 during differentiation of key cells were heterogeneous, potentially reflecting their distinct roles in cell differentiation. RPL14 and SMARCD3 likely function in different capacities during this process, influencing the differentiation trajectories of macrophages and neutrophils in ARDS.

### 3.12 High expression of SMARCD3 and TCN1 in LPS induced murine model of acute lung injury

**Figure 12:**
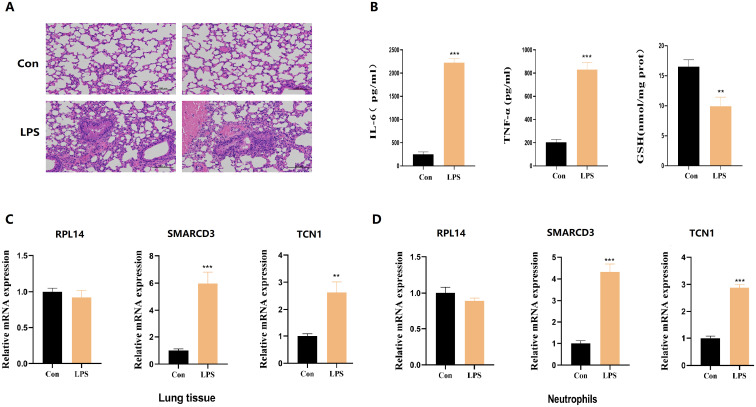
The expression levels of hub ARDS-ARDEGs (RPL14, SMARCD3, TCN1), inflammatory cytokines and GSH in murine acute lung injury models. **(A)** Histopathological images of lung tissues in LPS-induced ALI mice and control group. Scale bar, 100 mm. **(B)** ELISA analysis of inflammatory cytokines and GSH from ALI mice. **(C)** RPL14 mRNA expression remained unchanged but SMARCD3/TCN1 mRNA expression increased in ALI mice lung tissue homogenate. **(D)** RPL14, SMARCD3, TCN1 mRNA expression in ALI mice peripheral blood neutrophils. n=3 in each group. The data are shown as mean ± standard deviation;**p<0.01, ***p<0.001 vs control group.

Building upon these results, we next sought to determine whether these effects could be recapitulated *in vivo*.C57BL/6J mice were stratified into control and LPS-induced acute lung injury groups, and the validity of the model was validated using pathological morphology and inflammation/oxidative stress indicators. Histopathological examination of lung tissues via HE staining revealed pronounced structural disruption of alveoli in murine model of acute lung injury, characterized by widespread thickening of alveolar septa (**Figure 12A**). Further quantification by ELISA showed markedly increased concentrations of proinflammatory cytokines IL-6 and TNF-α, coupled with a significant reduction in GSH levels in lung tissue homogenates from LPS-induced acute lung injury mice relative to controls (**Figure 12B**). Subsequent RT-qPCR analysis demonstrated that RPL14 mRNA expression remained unchanged in both lung tissue homogenates and peripheral blood neutrophils of LPS-induced acute lung injury mice. In contrast, SMARCD3 and TCN1 transcript levels were significantly elevated in both lung tissue (**Figure 12C**) and neutrophils (**Figure 12D**) compared to control.

### 3.13 Distinct roles of RPL14, SMARCD3 and TCN1 in regulating mitochondrial function and inflammation

**Figure 13:**
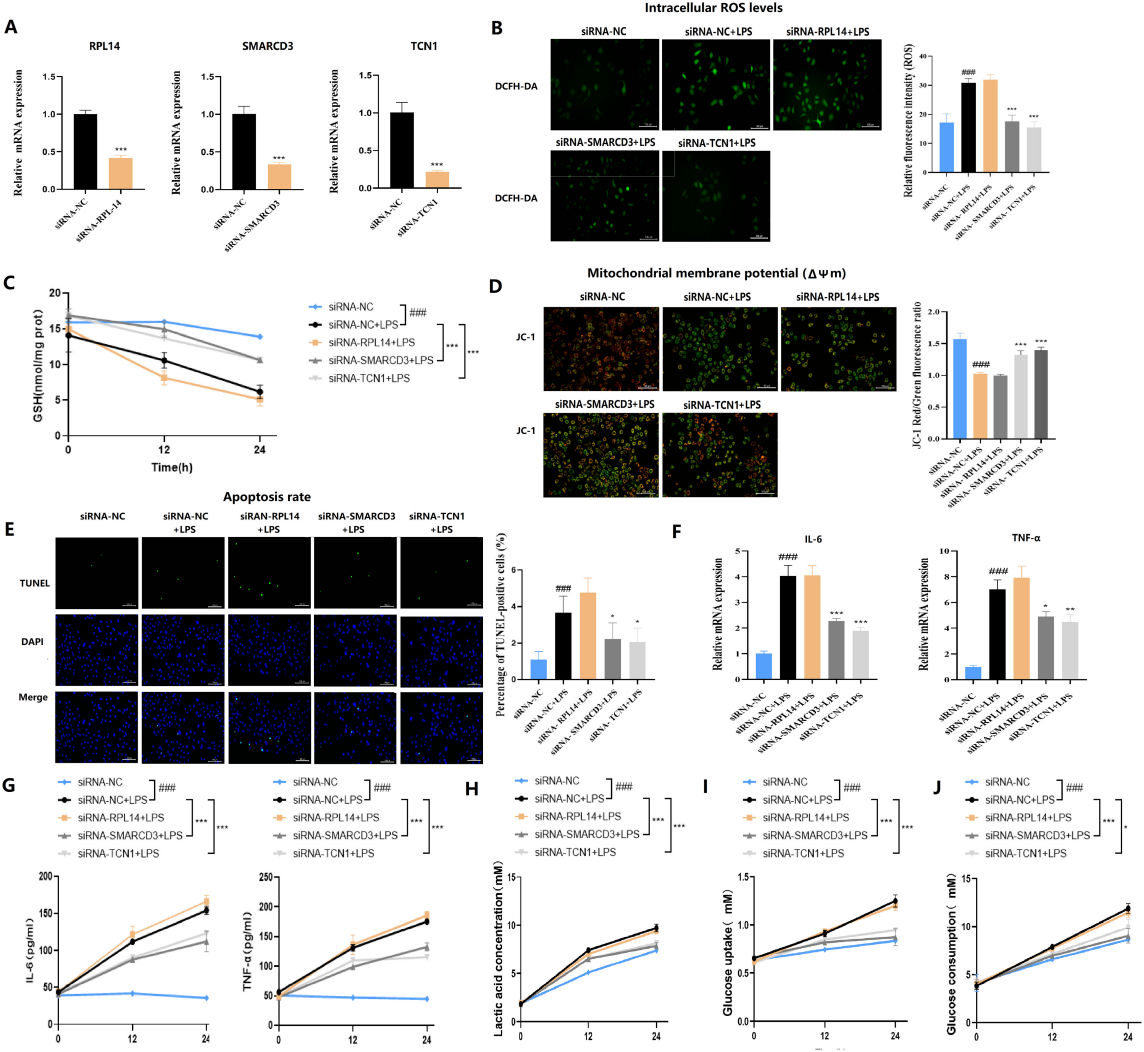
Functional validation of hub ARDS-ARDEGs (RPL14, SMARCD3, TCN1) in THP-1-derived macrophages. **(A)** siRNA-mediated silencing reduced the expression levels of the corresponding target genes in THP-1 cells. **(B)** SMARCD3/TCN1 silencing reduced ROS production with LPS stimulation. **(C)** ELISA analysis of GSH. **(D)** SMARCD3/TCN1 silencing led to increased mitochondrial membrane potential with LPS stimulation. **(E)** TUNEL assay showed SMARCD3/TCN1 silencing suppressed apoptotic activity. **(F)** Inflammatory cytokine mRNA expression. **(G)** ELISA of cytokine secretion levels. **(H)** Reduction in lactate production upon SMARCD3 or TCN1 knockdown. **(I)** Impaired glucose uptake following SMARCD3 or TCN1 silencing. **(J)** Decreased glucose consumption in SMARCD3- or TCN1-deficient cells. ###p<0.001 vs siRNA-NC group;*p<0.05, **p<0.01, ***p<0.001 vs siRNA-NC+LPS group.

To decipher the cellular mechanisms underlying the observed lung injury, we next performed *in vitro* experiments. THP-1 cells were first transfected with siRNA targeting RPL14, SMARCD3, or TCN1, then stimulated with LPS (**Figure 13A**). ROS detection (**Figure 13B**) revealed that RPL14 silencing did not affect mitochondrial membrane potential and concomitantly showed no change in JC-1 staining, with TUNEL assay further confirming no significant effect on cellular apoptosis. In contrast, both SMARCD3 and TCN1 silencing led to reduced ROS production and increased mitochondrial membrane potential, accompanied by suppressed apoptotic activity. Compared to the control group, GSH levels were significantly increased by SMARCD3 and TCN1 silencing after LPS stimulation (**Figures 13C-E**). RT-qPCR analysis revealed that RPL14 interference did not affect IL-6 or TNF-α mRNA expression, whereas SMARCD3 and TCN1 knockdown significantly reduced both cytokines (**Figure 13F**). Consistently, ELISA measurements showed RPL14 silencing had no notable effects on IL-6/TNF-α secretion; however, SMARCD3 and TCN1 silencing markedly inhibited IL-6/TNF-α secretion (**Figure 13G**). In terms of alterations in glucose metabolism, silencing SMARCD3 or TCN1, but not RPL14, decreased lactate production, glucose uptake and glucose consumption compared to the control group. (**Figures 13H-J**).

### 3.14 CsA inhibition of LPS-induced macrophage injury is associated with downregulation of SMARCD3

**Figure 14:**
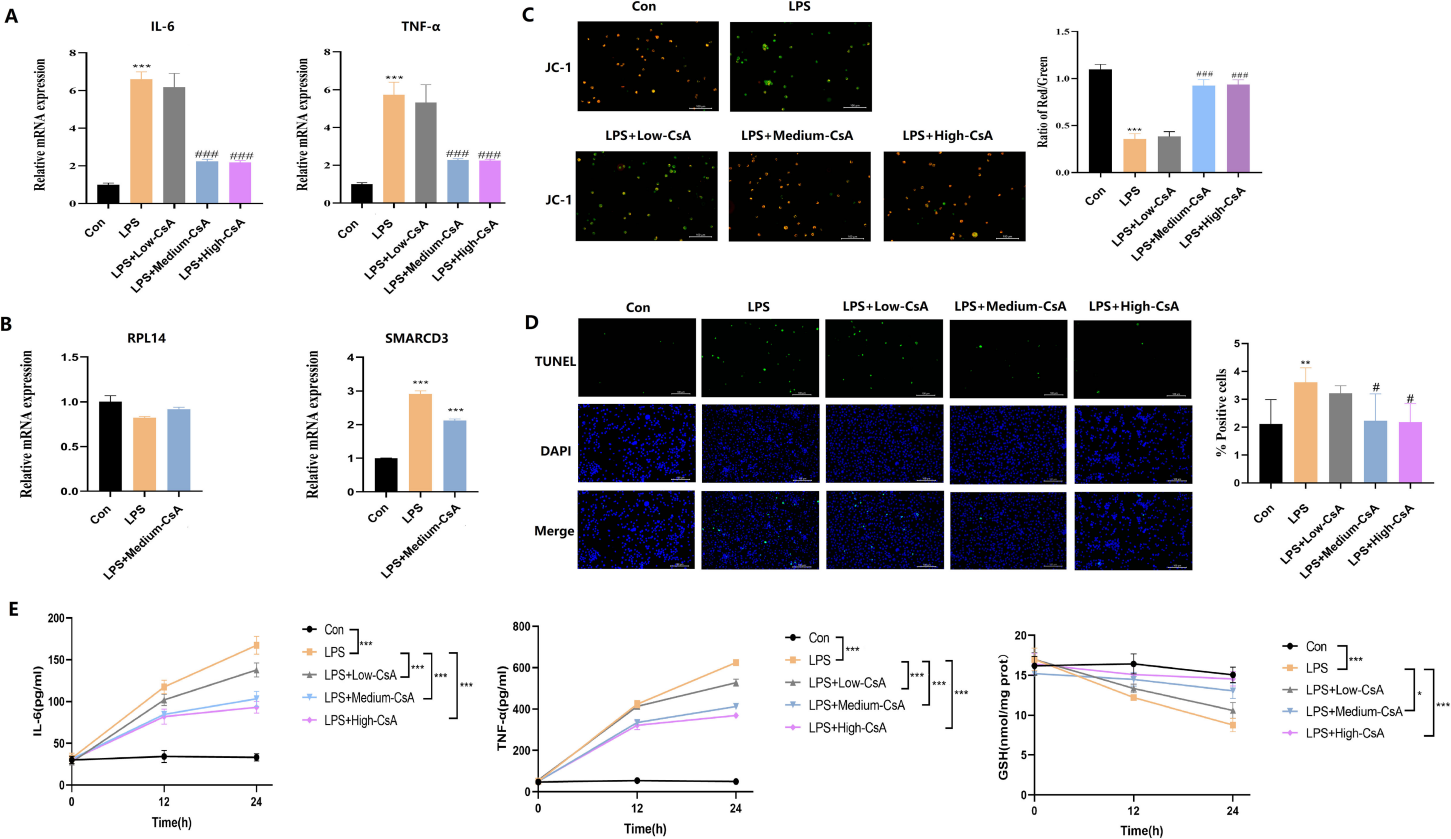
CsA reversed LPS-induced mitochondrial damage, inflammation, and oxidative stress in macrophages. **(A)** LPS-induced IL-6/TNF-a upregulation was attenuated by medium/high-dose CsA. **(B)** LPS stimulation enhances SMARCD3 expression (but not RPL14) in macrophages, while CsA administration reverses this LPS-induced SMARCD3 upregulation. **(C)** JC-1 assay. Medium- to high-concentration CsA dose-dependently reversed the LPS-induced reduction in mitochondrial membrane potential. **(D)** TUNEL assay. LPS-induced apoptosis was dose-dependently mitigated by medium/high-dose CsA. **(E)** Inflammatory markers and redox status. LPS triggered IL-6/TNF-a overproduction and GSH depletion, all dose-dependently attenuated by CsA (maximal efficacy at medium/high doses). The data are shown as mean ± standard deviation; *p<0.05, **p<0.01, ***p<0.001 vs control group; #p<0.05, ###p<0.001 vs LPS group.

To further investigate the role of CsA in LPS-induced macrophages, we differentiated THP-1 cells into macrophages with PMA (63). We measured cytokine expression by RT-qPCR, the result showed that LPS stimulation significantly upregulated IL-6 and TNF-α mRNA, while medium/high-dose CsA attenuated this upregulation (**Figure 14A**). Notably, the inhibitory effects of CsA extended beyond cytokine modulation to transcriptional regulators. LPS-treated macrophages exhibited unchanged RPL14 mRNA but significantly increased SMARCD3 expression. The LPS+CsA group showed no alteration in RPL14 versus LPS-only group, but displayed significant SMARCD3 downregulation (**Figure 14B**). These findings indicate that LPS stimulation enhances SMARCD3 expression in macrophages, while CsA administration reverses this LPS-induced SMARCD3 upregulation.

JC-1 (**Figure 14C**) and TUNEL assay (**Figure 14D**) revealed that LPS stimulation significantly reduced mitochondrial membrane potential and induced apoptosis in macrophages, effects that were dose-dependently reversed by medium/high-concentration CsA treatment. Concurrently, LPS triggered pro-inflammatory cytokine overproduction (IL-6, TNF-α) and depleted intracellular GSH, all of which were attenuated by CsA in a concentration-dependent manner. Notably, all CsA-treated groups exhibited significantly reduced cytokine secretion and restored GSH levels versus LPS-only controls, with maximal efficacy at medium/high doses (**Figure 14E**). These data demonstrate that CsA mitigates LPS-induced macrophage injury, and its protective effects are associated with downregulation of SMARCD3.

## 4 Discussion

The interplay between directional immune cell migration and metabolic reprogramming is pivotal in ARDS. Metabolic alterations not only impact immune cell function but also regulate their migration toward damaged lung tissue (66). For example, metabolic by-products can modify immune cells’ response to chemical signals, thereby affecting their migratory capacity (67). This relationship suggests that targeting these processes could be a viable strategy to control inflammation and mitigate lung damage in ARDS. This study identified three biomarkers (RPL14, SMARCD3, TCN1) through machine learning algorithms and explored their potential mechanisms in ARDS *via* ANN model construction, enrichment analysis, regulatory network construction, and drug prediction analysis. Key cells were subsequently identified using single-cell data, and the expression of biomarkers was examined at the cellular level, offering new insights into the pathogenesis of ARDS.

SMARCD3 (SWI/SNF-Related Matrix-Associated Actin-Dependent Regulator of Chromatin Subfamily D Member 3) plays a pivotal role in chromatin remodeling and regulates essential cellular processes, including differentiation, proliferation, and apoptosis. Its dysfunction is associated with a range of diseases. In cancer, alterations in chromatin structure and gene expression disrupt cell cycle regulation and modify the activity of proto-oncogenes or tumor suppressor genes (68, 69). Although SMARCD3 has not been directly studied in ARDS, chromatin remodeling is well-established as a critical factor in controlling the inflammatory responses central to ARDS pathogenesis. In infectious diseases, including viral infections like influenza and Coronavirus Disease 2019(COVID-19), SMARCD3 is involved in regulating the immune response (70, 71). Additionally, chromatin remodeling influences the epigenetics of immune cells in sepsis, suggesting an indirect link between SMARCD3 and ARDS development (68, 69, 72). Furthermore, immune cell metabolic requirements and pathways undergo significant changes during different activation states. For instance, lipid metabolic reprogramming in CD8+ T cells is crucial for anti-tumor immunity. As a chromatin remodeling factor, SMARCD3 may modulate immune cell metabolic reprogramming by regulating key metabolic genes, ultimately affecting their immunological functions (73). Our study observed that upregulation of SMARCD3 in ARDS may drive macrophages and neutrophils toward a glycolysis-dependent M1/N1-like polarization state. This metabolic reprogramming not only meets the heightened energy demands and biosynthetic precursor requirements for rapid cellular proliferation and activation, but also promotes reactive oxygen species (ROS) generation and the release of proinflammatory cytokines such as IL-6 and TNF-α. In summary, SMARCD3 likely serves as a crucial molecular regulator of inflammatory responses in ARDS by reprogramming metabolic pathways in immune cells.

TCN1 (Transcobalamin 1) is a critical transporter of vitamin B12 in the bloodstream, ensuring the delivery of vitamin B12 to tissues and cells, thus supporting normal metabolic and physiological functions (74, 75). Dysfunction of TCN1 can impair vitamin B12 transport, leading to metabolic disturbances. Vitamin B12 is integral to cellular metabolism, and as a transport protein, TCN1 may influence vitamin B12 levels in immune cells, thereby impacting their metabolic processes (76). In inflammatory conditions like ulcerative colitis, TCN1 expression can be altered by inflammation (74). Although TCN1 has not been directly studied in ARDS, vitamin B12 metabolism is crucial for cellular function and immune responses, and TCN1 may therefore indirectly affect the progression of ARDS. In infectious diseases such as COVID-19, TCN1 has been linked to poor outcomes, with vitamin B12 deficiency associated with a worse prognosis (77). Altered TCN1 levels have also been observed in sepsis, likely due to its role in immune modulation and cellular metabolism (77). Furthermore, TCN1 is implicated in autoimmune diseases, where it may help regulate inflammatory responses (74, 75, 77). This study found that TCN1 was significantly upregulated in ARDS samples, and after silencing TCN1, the intracellular GSH level increased and the secretion of inflammatory factors decreased. This result suggests that TCN1 may affect the activation state of immune cells and the output of inflammation by regulating the vitamin B12-dependent metabolic pathway: its abnormal expression may reshape the methylation metabolic efficiency and antioxidant capacity of immune cells, and ultimately participate in the pathological process of ARDS. In summary, TCN1, as a key molecule connecting vitamin B12 metabolism and immune inflammation, provides a new perspective for understanding the cross-regulation mechanism of immunity and metabolism in ARDS.

RPL14 (Ribosomal Protein L14) is essential for ribosome function and plays a role in cell growth, proliferation, and cancer progression (78). Mutations in the RPL14 gene can disrupt protein synthesis, leading to developmental disorders. RPL14 also influences immunity, as it is often disrupted during viral infections (e.g., influenza, COVID-19), where viruses hijack ribosomal proteins to replicate and evade immune defenses (79). RPL14 is involved in ribosome construction, which is directly related to protein synthesis and energy metabolism. Thus, RPL14 may regulate the energy metabolism pathways of immune cells, such as glycolysis and OXPHOS, by influencing protein synthesis (80). Additionally, RPL14 is linked to immune dysfunction in sepsis, a leading cause of death. In cancer, elevated levels of RPL14 correlate with tumor growth and aggression (81).

SMARCD3 and TCN1, not RPL14, act as key molecular nodes linking inflammatory signaling (LPS/TLR4) to metabolic reprogramming. Silencing SMARCD3 or TCN1 leads to metabolic alterations characterized by reduced glucose uptake, glucose consumption and lactate production, thereby inhibiting glycolytic flux. At the functional level, this suppression prevents polarization toward a pro-inflammatory phenotype and enhances antioxidant capacity.

The RT-qPCR results from clinical patients with ARDS confirmed that SMARCD3 and TCN1 are highly expressed in ARDS, consistent with the bioinformatics analysis. However, RPL14 expression showed a decreasing trend in ARDS samples, though this difference was not statistically significant, likely due to the limited sample size. GSEA analysis revealed significant enrichment of 82, 89, and 69 pathways for RPL14, SMARCD3, and TCN1, respectively. Notably, the ribosome pathway was strongly associated with both RPL14 and SMARCD3, while chemokine, B cell receptor, and T cell receptor signaling pathways were significantly associated with both SMARCD3 and TCN1.

The pathways associated with SMARCD3, TCN1, and RPL14 converge on key processes critical in ARDS, including: (1) Immune Cell Recruitment and Activation: Chemokine and receptor signaling pathways, such as chemokine, B cell receptor, and T cell receptor pathways, are essential for directing immune cells to the lung and regulating their function. Dysregulation of these pathways can lead to excessive inflammation and tissue damage (82–84). (2) Inflammatory Signaling: TLR and neurotrophin signaling pathways underline the role of innate immunity and neuroimmune interactions in ARDS. These pathways contribute to the hyperinflammatory state and systemic effects associated with ARDS (82, 85). (3) Cellular Stress and Repair: Ribosome and spliceosome pathways highlight the importance of cellular stress and repair processes. Dysregulation of these pathways can impair lung tissue repair, exacerbating injury (82).

The biomarkers SMARCD3, TCN1, and RPL14 are linked to critical pathways involved in immune regulation, inflammation, and cellular repair in ARDS. Their roles in chemokine signaling, receptor signaling, and ribosomal function suggest they may contribute to the dysregulated immune response and tissue damage observed in ARDS. Further research is necessary to elucidate their specific mechanisms and potential as therapeutic targets.

In this study, a comprehensive molecular regulatory network involving miRNAs, lncRNAs, and TFs was constructed to investigate the potential mechanisms underlying ARDS. The network highlights interactions between two key biomarkers, RPL14 and SMARCD3, and their regulatory molecules. TCN1 appears to play a less prominent role in the network due to the lack of predicted miRNA interactions.

MiRNA-Mediated Regulation of Biomarkers: MiRNAs typically bind to the 3’ untranslated region (3’UTR) of target mRNAs, leading to mRNA degradation or translational repression (86). In ARDS, dysregulated miRNAs have been shown to modulate inflammatory pathways, oxidative stress, and cell apoptosis—key processes in ARDS development (87, 88). For instance, miR-155 and miR-146a, which are commonly dysregulated in ARDS, target genes involved in NF-κB signaling and cytokine production, thus contributing to the inflammatory response (87).

Based on the above information (68, 69, 74, 75, 79, 89), this study hypothesizes the following potential mechanisms: Dysregulation of RPL14 could impair ribosomal function, leading to defective protein synthesis and heightened susceptibility to cellular damage in ARDS (79, 89). SMARCD3 may be targeted by miRNAs, which regulate gene expression and chromatin structure. Altered SMARCD3 expression could disrupt the transcriptional regulation of inflammatory genes, thereby exacerbating ARDS pathology (68, 69). The absence of predicted miRNAs targeting TCN1 suggests that its regulation may occur at other levels, such as transcriptional or post-translational modifications (90). TCN1, as a vitamin B12-binding protein, may influence ARDS through mechanisms independent of miRNA regulation, such as modulation of oxidative stress or immune responses.

KLF9 (Krüppel-like factor 9), a TF that targets both RPL14 and SMARCD3, plays a key role in regulating cell differentiation, inflammation, and stress responses. In chronic obstructive pulmonary disease (COPD), KLF9 modulates airway inflammation by controlling pro-inflammatory cytokines and antioxidant genes (91), suggesting a similar function in ARDS due to the shared inflammatory mechanisms between the two conditions. KLF9 has also been implicated in airway remodeling and inflammation in benign tracheal stenosis (92), as well as in asthma and pulmonary fibrosis, where it regulates epithelial-mesenchymal transition (EMT) and fibrotic responses—processes particularly relevant to ARDS, especially during the fibroproliferative phase. By regulating RPL14 and SMARCD3, KLF9 may influence ribosomal function and chromatin remodeling, affecting inflammatory gene expression and protein synthesis under stress conditions in ARDS. This regulation of RPL14 and SMARCD3 by KLF9 could modulate cellular responses to injury and inflammation in ARDS, with KLF9-mediated changes in SMARCD3 expression potentially altering inflammatory gene regulation and its impact on protein synthesis through RPL14 under stress.

In drug prediction, molecular docking is a widely used computational method, offering a preliminary assessment of the binding ability between drugs and targets. Despite its limitations, drug prediction remains a critical step in drug development. Three drugs—tetradioxin (CTD 00006848), selenium (CTD 00006731), and CsA (CTD 00007121)—are notable for their ability to target multiple biomarkers simultaneously. Although direct evidence for tetradioxin in ARDS is lacking, its dual targeting of SMARCD3 and TCN1 suggests it may regulate inflammatory and metabolic pathways pertinent to ARDS. Both chromatin remodeling and metabolic dysregulation have been implicated in respiratory diseases like COVID-19 and COPD (93, 94). Selenium has been extensively studied in the context of respiratory diseases, including ARDS and COVID-19. Selenium deficiency is associated with worsened severity of respiratory infections and inflammation, while supplementation has been shown to alleviate oxidative stress and improve ARDS prognosis (94, 95). Its role in immune regulation and antioxidant defense positions selenium as a promising agent for ARDS treatment, particularly for patients with comorbidities such as asthma or vitamin D deficiency (96). CsA has been explored in ARDS and other inflammatory lung diseases for its ability to suppress cytokine storms and reduce lung injury. Its immunosuppressive properties make it a potential therapeutic option for ARDS, especially in hyperinflammatory cases (97). CsA has also been studied in COVID-19, where cytokine storms and immune dysregulation play a central role in disease progression (94). These drugs may also offer broader therapeutic applications in systemic diseases with similar pathogenesis, such as COVID-19, COPD, and asthma, where inflammation, oxidative stress, and immune dysregulation are key drivers of disease progression (94, 95). Although drug prediction analysis is primarily based on computational results, the drugs identified provide valuable leads for further experimental research, potentially accelerating the drug development process.

Single-cell data offer in-depth insights into cellular heterogeneity, enabling the identification of distinct cell types and states at the individual level. This is particularly important in ARDS, a condition characterized by diverse cell types and complex mechanisms. Single-cell sequencing can unveil specific immune cell subsets and their interactions in ARDS. Analysis of the GSE180578 dataset through single-cell sequencing identified eight distinct cell types, with macrophages, neutrophils, and endothelial cells being enriched in ARDS samples (66, 98, 99). Dynamic intercellular interactions were observed during the pathogenesis of ARDS, emphasizing the complex nature of immune responses. Specifically, 16 subtypes of macrophages and 9 subtypes of neutrophils were identified, highlighting the functional diversity within these populations. This level of detail is beyond the reach of conventional bulk transcriptomics (100) and provides valuable insights into the specific role of immune cells in ARDS progression.

Our findings highlight the critical role of macrophages and neutrophils in ARDS. Increased macrophage activity, consistent with their dual roles in inflammation and tissue repair (66, 98), likely contributes to the upregulation of RPL14 and SMARCD3 expression in certain subtypes. Neutrophils’ robust communication network may facilitate ARDS progression through the formation of NETs and IL-10-mediated inflammatory responses (78, 80). The differential expression of RPL14 and SMARCD3 suggests their involvement in metabolic or transcriptional regulation during cellular activation (100–102). While scRNA-seq of blood samples has its limitations—since blood does not fully reflect the local pathological and physiological changes in the lungs during ARDS—it still provides crucial information about the systemic immune response and offers valuable clues for understanding immune dynamics in ARDS.

Pseudo-temporal trajectory analysis revealed distinct expression patterns of RPL14 and SMARCD3 during macrophage and neutrophil differentiation. In macrophages, RPL14 peaks early and then declines, potentially contributing to early inflammatory responses (98). In contrast, SMARCD3 increases gradually, suggesting its role in regulating anti-inflammatory or metabolic adaptations during later stages. In neutrophils, RPL14 remains elevated, supporting sustained inflammatory processes, while SMARCD3 peaks during mid-differentiation, likely promoting functional maturation (99, 100). These expression profiles support the hypothesis that metabolic reprogramming modulates immune cell behavior in ARDS, with RPL14 promoting glycolysis in early macrophage activation and SMARCD3 facilitating the transition to an anti-inflammatory state (101, 102).

This study has several limitations. First, the specific mechanisms of biomarkers in ARDS and the precise actions of the predicted drugs require further experimental validation. Second, the biomarkers were analyzed only in whole blood samples, not in lung tissue or bronchoalveolar lavage fluid, which are more directly related to ARDS pathophysiology. Biomarker expression may also vary over time, yet the pseudotime analysis focused only on differentiation status rather than disease progression. To address these limitations, subsequent studies will integrate cellular and animal models, combined with metabolic analysis (such as Seahorse XF Analyzer assays), to further investigate the mechanisms of immune-metabolic biomarkers in ARDS and predict drug action pathways. Moreover, the RT-qPCR verification sample size will be expanded to include patients at different disease stages and levels of severity. Other techniques, such as Western blotting, will be added for further validation to enhance the reliability of the results. Flow cytometry will be used to sort different types of immune cells from peripheral blood for the detection of RPL14, SMARCD3 and TCN1 expression levels; Additionally, single-cell data will be collected from lung tissue or bronchoalveolar lavage fluid and integrated with clinical data to create a more comprehensive map of the local immune environment.

This approach will deepen our understanding of the disease mechanisms of ARDS and provide a more robust theoretical foundation for clinical diagnosis and treatment.

## 5 Conclusion

In conclusion, this study identified candidate biomarkers—RPL14, SMARCD3, and TCN1—linked to immune cell activity and metabolic reprogramming in ARDS, and developed an ANN model. Functional enrichment analysis highlighted the biological pathways through which these biomarkers influence ARDS pathogenesis. Single-cell analysis further explored the cellular expression of these biomarkers. Both *in vitro* and *in vivo* experiments demonstrated that hub ARDS-ARDEGs (SMARCD3 and TCN1,but not RPL14) significantly affected mitochondrial function, oxidative stress, apoptosis, glucose metabolism and inflammatory cytokine expression, offering new insights into potential mechanisms underlying ARDS and providing valuable information for optimizing clinical treatment strategies. It is noteworthy that while RPL14 demonstrated predictive value in transcriptomic analysis and ANN modeling, its functional role in ARDS requires further validation.

## Data Availability

The datasets presented in this study can be found in online repositories. The names of the repository/repositories and accession number(s) can be found in the article/**Supplementary Material**.

## Supplementary material

Supplementary Table 1 List of 1804 PRGs.

Supplementary Table 2 Cell type markers.

Supplementary Table 3 Primer sequences for biomarkers andβ-actin.

Supplementary Table 4 siRNA sequence.

Supplementary Table 5 The top 10 immune cells in terms of infiltration abundance.

Supplementary Table 6 35 differential immune cells.

Supplementary Table 7 Gene Ontology analysis results.

Supplementary Table 8 KEGG pathway enrichment analysis results.

Supplementary Table 9 Gene set enrichment analysis (GSEA) results for RPL14.

Supplementary Table 10 Gene set enrichment analysis (GSEA) results for SMARCD3.

Supplementary Table 11 Gene set enrichment analysis (GSEA) results for TCN1.

Supplementary Table 12 Drug response analysis.

Supplementary Table 13 The binding energies of drug-gene.

## Glossary

3’UTR: 3’ untranslated region
ANN: An artificial neural network
ARDS: Acute respiratory distress syndrome
ALI: Acute lung injury
AUC: Area under the curve
CsA: cyclosporine A
COPD: chronic obstructive pulmonary disease
COVID-19: Coronavirus Disease 2019
DEGs: Differentially expressed genes
DESeq2: Differential Expression Sequencing analysis
ELISA: Enzyme-Linked Immunosorbent Assay
EMT: Epithelial-Mesenchymal Transition
FAO: Fatty acid oxidation
GGI: Gene-gene interaction
GO: Gene ontology
GSEA: Gene set enrichment analysis
GSH: Glutathione
HVGs: High-variant genes
HE: Hematoxylin and eosin
IL-1β: Interleukin-1β
IL-6: Interleukin-6
KEGG: Kyoto Encyclopedia of Genes and Genomes
KLF9: Krüppel-like factor 9
LASSO: Least absolute shrinkage and selection operator
LPS: Lipopolysaccharide
MRRGs: Metabolic reprogramming-related genes
MSigDB: Molecular Signatures Database
NETs: Neutrophil extracellular traps
OXPHOS: Oxidative phosphorylation
PBS: Phosphate-buffered saline
PCA: Principal component analysis
PCs: Principal components
PMA: Phorbol 12-myristate 13-acetate
PPI: Protein-protein interaction
PPP: Pentose phosphate pathway
ROC: Receiver operating characteristic
RPL14: Ribosomal protein L14
RT-qPCR: Reverse transcription-quantitative polymerase chain reaction
SMARCD3: Sw/snf-related matrix-associated actin-dependent regulator of chromatin subfamily d member 3
SVM-RFE: Support vector machine recursive feature elimination
TCN1: Transcobalamin 1
TF: Transcription factor
THP-1: Tohoku Hospital Pediatrics-1
TNF-α: Tumor necrosis factor-α
UMAP: Uniform manifold approximation and projection
WGCNA: Weighted gene co-expression network analysis.
