# Supplementary material for: Decoding immune-metabolic crosstalk in ARDS: a transcriptomic exploration of biomarkers, cellular dynamics, and therapeutic pathways
Source: Front Immunol. 2025 Oct 27;16:1615748. doi: 10.3389/fimmu.2025.1615748 (PMC12597791; doi:10.3389/fimmu.2025.1615748)
Supplement: Supplementary Table 5 — The top 10 immune cells in terms of infiltration abundance. [file Table5.doc]

**Additional File 5 The top 10 immune cells in terms of infiltration abundance**

| **Cell** | **Average** |
| --- | --- |
| Neutrophils | 4524.47805505614 |
| Monocytes | 3608.41853433289 |
| Erythrocytes | 3304.60079931782 |
| MPP | 2609.52319599194 |
| Eosinophils | 2266.08248076928 |
| Megakaryocytes | 2147.37123281944 |
| GMP | 2118.96490884621 |
| iDC | 1952.51410810128 |
| CD8+ Tem | 1912.46386074595 |
| pDC | 1869.11652537653 |
| Neutrophils | 4524.47805505614 |
