# Supplementary material for: Decoding immune-metabolic crosstalk in ARDS: a transcriptomic exploration of biomarkers, cellular dynamics, and therapeutic pathways
Source: Front Immunol. 2025 Oct 27;16:1615748. doi: 10.3389/fimmu.2025.1615748 (PMC12597791; doi:10.3389/fimmu.2025.1615748)
Supplement: Supplementary Table 13 — The binding energies of drug-gene. [file Table13.doc]

**Additional File 13** The binding energies of drug-gene

| **Ligand** | **Protein** | **Vina score** | **Center(x,y,z)** | **Docking size(x,y,z)** |
| --- | --- | --- | --- | --- |
| RPL14-mesalazine | RPL14 | -4.5 | -4, -3, -1 | 17, 17, 17 |
| TCN1-arbutin | TCN1 | -7.1 | -22, 38, 4 | 20, 20, 20 |
| SMARCD3-Tetradioxin | SMARCD3 | -6.8 | -15, -3, 10 | 20, 20, 20 |
